# Geometry‐Encoded Soft Strain Sensing via Liquid‐Metal Transmission Lines

**DOI:** 10.1002/advs.77839

**Published:** 2026-09-16

**Authors:** Zhang Liu, Fengdeng Jin, Wenxuan Shi, Meng She, Ziqi Meng, Miao Fang, Yixin Zhang, Qianwen Dong, Wandi Yang, Lei Shi, Xujia Zhao, Feng Zhou, Bin Yao

**Affiliations:** ^1^ School of Aerospace Science and Technology Xidian University Xi'an China; ^2^ Honghui Hospital Xi'an Jiaotong University Xi'an China; ^3^ School of Electronic Engineering Xidian University Xi'an China; ^4^ School of Mechano‐Electronic Engineering Xidian University Xi'an China; ^5^ School of Optoelectronic Engineering Xidian University Xi'an China

**Keywords:** decoupling sensing, flexible electronics, liquid metals, strain sensor

## Abstract

Reliable strain sensing in soft systems remains challenging under mechanically complex conditions, where compression, folding, transverse deformation, and environmental perturbations often interfere with tensile‐strain readout. Existing flexible strain sensors typically rely on amplitude‐based electrical responses and therefore frequently require calibration, compensation, or signal reconstruction to isolate axial deformation. Here, we present a soft liquid‐metal transmission‐line sensor that directly encodes axial elongation into the time‐of‐flight of an electromagnetic pulse. Because the readout is governed by the total propagation path length, deformation modes that do not alter this path—including localized compression, folding, and biaxial transverse strain—produce negligible influence on the measured signal. The sensor exhibits linear strain response over a wide working range up to 400% strain, together with a strain‐range–independent length resolution of 10 mm. Owing to its geometry‐governed mechanism, the device enables self‐referenced and calibration‐free strain measurement with strong inter‐device consistency, while maintaining stable operation under large pre‐strain, cyclic loading, and irreversible deformation. Reliable sensing is further demonstrated on curved surfaces, wearable systems, inflatable structures, pneumatic artificial muscles, and task‐level clinical tourniquet monitoring under dynamically varying deformation conditions. This work provides a robust and reconstruction‐free strategy for strain sensing in soft electronics, wearable systems, and clinical healthcare.

## Graphical abstract

This study presents a liquid‐metal transmission‐line strain sensor that achieves intrinsic decoupling by encoding axial elongation into propagation delay. The time‐domain readout remains stable under complex off‐axis. This enables reliable, repeatable, real‐time elongation measurement in dynamic, multiaxial loading environments for wearable electronics, soft robotics, and structural health monitoring.

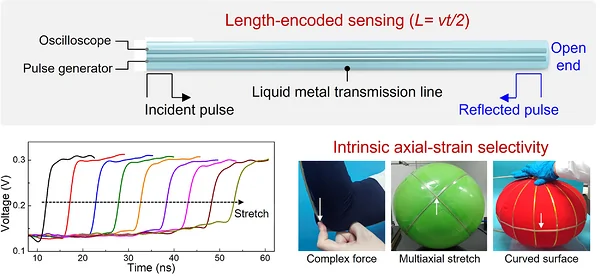

## 1 Introduction

Flexible and stretchable strain sensors are essential components in wearable electronics, soft robotics, and human–machine interfaces, where accurate measurement of tensile deformation is critical for reliable system performance [1, 2]. In practical scenarios, these systems are subjected to complex, multidirectional mechanical stimuli, including stretching, compression, bending, and folding, often occurring simultaneously [3, 4, 5, 6]. Soft strain sensors placed in such settings inevitably experience mixed mechanical inputs, and their signals are influenced by multiple deformation modes, rather than reflecting solely the axial stretch of interest. This can lead to measurement errors if the sensor lacks decoupling capabilities [7, 8]. In this context, a sensing mechanism that selectively encodes axial elongation into an independent and minimally coupled signal parameter would provide a fundamentally more reliable and accurate measure of strain under complex conditions.

**FIGURE 1:**
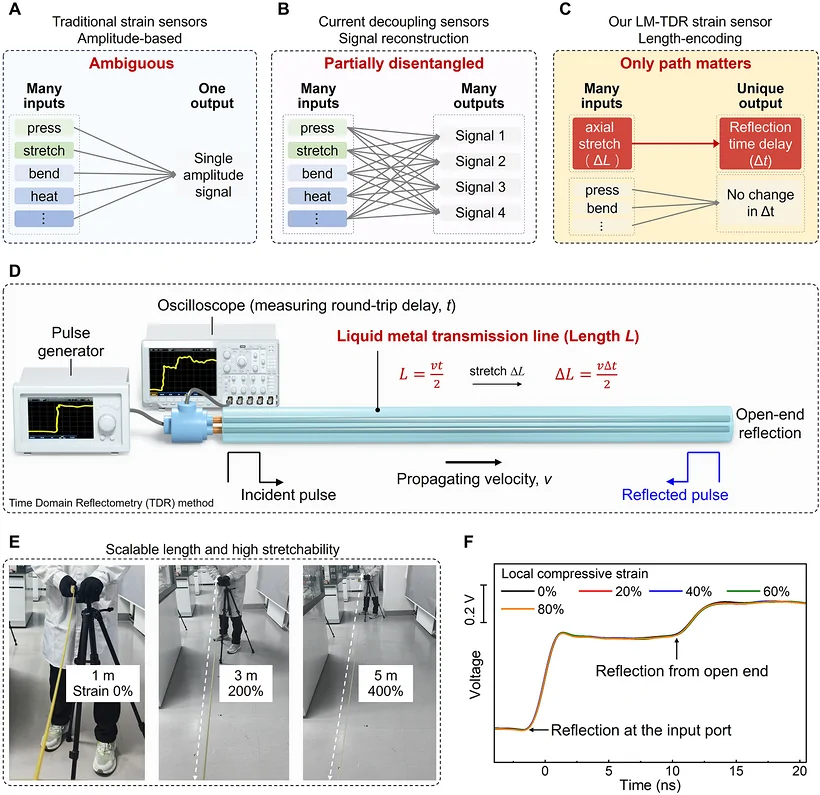
The proposed length‐encoded strain sensing: A Conventional soft sensors map mixed mechanical inputs to a single amplitude signal, resulting in ambiguous, non‐decoupled responses. B Existing decoupling strategies rely on multiple outputs for indirect inference, achieving only partial disentanglement. C The LM–TDR sensor maps axial elongation to propagation length and thus to reflection delay. Perturbations that do not alter the propagation path do not affect the delay, enabling intrinsic parameter‐level decoupling. D Working principle of our LM‐TDR sensor. A pulse is launched into the LM transmission line, and the oscilloscope records the round‐trip delay *t* of the reflection from the open end. The line length is determined as *L* = *vt*/2, with elongation‐induced changes Δ*L* = *v*Δ*t*/2. E Large stretchability of an LM–TDR sensor. F Responses remain unchanged under localized transverse compression at the midpoint.

Common soft strain sensors are typically designed to measure uniaxial strain along a known direction, from which the applied deformation can be inferred via electrical signals (e.g., resistance, capacitance, current, or voltage) [9, 10, 11]. However, under complex loading conditions, multiple mechanical inputs are mapped onto a single output signal, resulting in an intrinsically ambiguous many‐to‐one transduction that lacks a unique inverse mapping (Figure 1A). As a result, these common soft sensors often fail to reliably isolate the intended strain from off‐axis deformations. Operation is further complicated by installation effects, such as curvature or pre‐tension introduced during mounting, which can induce baseline shift and necessitate post‐installation zeroing and calibration [12]. In addition, the soft polymer matrices inherently exhibit viscoelastic creep, causing gradual signal shifts under repeated or prolonged deformation [13], and their electrical properties are also sensitive to temperature [14], introducing additional long‐term drift [15].

To address the intrinsic ambiguity of many‐to‐one transduction, most existing approaches adopt a many‐to‐many paradigm, in which multiple mechanical stimuli are mapped onto multiple electrical outputs (Figure 1B). This expanded readout space allows partial disentanglement of deformation modes via signal comparison, reconstruction, or classification [4, 6, 8, 16]. These include multi‐channel sensor arrays [14, 17, 18, 19, 20], spatially distributed architectures [21, 22, 23, 24, 25], stimulus‐selective materials [26, 27, 28, 29], structure enhancing the sensitivity to elongation [30, 31], or data‐driven signal reconstruction [9, 32]. While these methods can partially improve discrimination, they remain fundamentally dependent on coupled transduction mechanisms, in which each output channel is still influenced by multiple mechanical inputs [3, 6, 31, 33, 34]. As a result, the relationship between measured signals and underlying deformation is typically indirect and non‐unique, requiring calibration, modeling, or machine‐learning‐based reconstruction. Consequently, the sensing performance often becomes highly scenario‐dependent and sensitive to variations in geometry, loading conditions, or material state, limiting robustness and general applicability in dynamically changing environments.

Electrical time‐domain reflectometry (TDR) offers a fundamental physical route to bypass these amplitude‐dependent limitations by inferring propagation distance directly from the round‐trip delay of an injected high‐speed electrical transient along a transmission line. Conventional TDR systems based on rigid metallic transmission lines have long been deployed for structural diagnostics [35, 36, 37, 38]. A seminal breakthrough was achieved by Leber et al. in integrating intrinsically deformable liquid‐metal (LM) transmission lines with TDR systems for multimodal sensing, marking a pivotal milestone in soft stretchable electronics [3]. This breakthrough spurred subsequent developments, including multi‐segment stretchable strain sensors and real‐time CMOS‐based TDR implementations [39, 40]. However, over the past six years, research in soft TDR sensing has predominantly focused on complex impedance‐discontinuity algorithms to reconstruct localized multi‐modal deformation profiles. Consequently, a profound physical capability inherent to transmission‐line electrodynamics has remained largely unexploited and unappreciated: its parameter‐level, intrinsic immunity to off‐axis mechanical perturbations when configured for global strain sensing. Although our preliminary work explored a single‐line LM configuration using high‐frequency phase delays [41], it was susceptible to stray circuit capacitance and required differential reference lines, obscuring the physical elegance of direct time‐of‐flight readout.

Here, we present a class of LM‐filled elastomeric conduit TDR sensors that harness this long‐overlooked physics to establish an intrinsic, parameter‐domain transduction between axial strain and signal time‐of‐flight (Figure 1C). By directly binding the transduction metric strictly to the total electromagnetic propagation path, tensile deformation modifies only the effective length, creating a direct, algorithm‐free one‐to‐one correspondence between axial strain and temporal delay. Crucially, mechanical perturbations that leave the propagation path unchanged—such as transverse compression, local folding, orthogonal stretching, or thermal variations—do not contaminate this encoded temporal parameter. In contrast to conventional flexible sensors that rely on geometry‐sensitive amplitude signals or complex multi‐parameter reconstruction, the proposed mechanism maps the target deformation onto a single, physically isolated observable, enabling highly linear, baseline‐free operation with natural sample‐to‐sample consistency and ease of deployment. We systematically validate this intrinsic decoupling principle across a series of mechanically demanding scenarios, including conformal length tracking on curved surfaces, pneumatic muscle actuation under large pre‐strain, cyclic mechanical conditioning, wearable joint‐angle monitoring under incidental contact, and multiaxial deformation configurations. These results establish LM–TDR sensing as a robust and versatile platform for selective tensile‐strain measurement in mechanically complex environments relevant to wearable electronics, soft robotics, and structural health monitoring.

## 2 Results and Discussion

### 2.1 Design Concept of the LM–TDR Sensor

Figure 1D illustrates the operating principle of our sensor. The stretchable transmission line, fabricated by filling LM (gallium‐based alloys [42, 43]) into elastomeric tubes, functions as a highly stretchable strain‐sensing element. When a voltage pulse is launched into the LM conduit, a strong reflection is generated at the open end due to impedance mismatch, while an oscilloscope simultaneously records the incident and reflected waveforms. The round‐trip delay *t* between incident and reflected signals is directly proportional to the effective line length, *L* = *vt*/2, where *v* is the signal propagation velocity and is treated as constant assuming the effective permittivity of the dielectric remains unchanged. Elongation‐induced length variations can then be obtained from the corresponding change in delay, Δ*L* = *v*Δ*t*/2, where Δ*L* denotes the applied elongation and Δ*t* the associated change in round‐trip delay.

The LM transmission line can be described by a distributed circuit model characterized by resistance (*R*), inductance (*L*), capacitance (*C)*, and conductance (*G*) per unit length (Figure S1). These parameters determine the characteristic impedance and govern electromagnetic signal transmission. Importantly, the propagation velocity is primarily set by the effective dielectric environment and remains approximately constant under mechanical deformation, provided the dielectric properties are unchanged. Under these conditions, the time‐of‐flight of the primary reflection depends solely on the total propagation path length. Tensile deformation increases this path length, leading to a proportional delay in the reflected signal. In contrast, localized perturbations such as compression or folding may introduce impedance discontinuities and generate secondary reflections, but do not change the total propagation path as long as electrical continuity is preserved [44]. Consequently, the arrival time of the primary reflection remains unchanged. This distinction between global path length and local impedance variations underlies the length‐encoded sensing mechanism: only deformation that modifies the propagation path contributes to the measured delay, whereas path‐invariant perturbations are intrinsically excluded.

To ensure stable propagation‐based sensing under large deformation, the transmission line was implemented using a parallel‐conductor LM architecture. Unlike single‐conductor designs that lack a dedicated ground return path and suffer from environmental coupling (where parasitic capacitances to surroundings fluctuate unpredictably), this twin‐line configuration forms a self‐contained, closed‐loop transmission network (Note S1). Consequently, the electromagnetic energy is strictly confined within the elastomer encapsulation layer, conferring high immunity against external parasitic interference and guaranteeing stable, deterministic delay‐length mapping under dynamic deformation. Coaxial structures, although offering well‐defined impedance in rigid systems, were not adopted due to their risk in mechanical instability under large strain, particularly the susceptibility of an outer LM sheath to structural disruption [45]. Parallel‐conductor LM transmission lines have been previously explored in stretchable radio‐frequency systems and are typically characterized in the frequency domain, demonstrating stable electromagnetic transmission under mechanical loading [46, 47, 48, 49]. However, such approaches primarily probe signal amplitude and spectral characteristics, and do not directly access propagation length as a geometry‐defined parameter. In contrast, our work employs TDR to extract the time‐of‐flight of pulses, enabling direct encoding of propagation length into a temporal observable.

While stretchable LM transmission lines have been previously explored in soft electronics—most notably by Leber et al. for multimodal deformation localization [3] and subsequently by others for multi‐segment sensing [39] and integrated CMOS readouts [40]—these foundational works primarily focused on complex impedance‐discontinuity reconstruction or multi‐peak tracking. In contrast, our approach redefines the LM–TDR platform by explicitly isolating global axial length from non‐axial mechanical perturbations, converting the time‐of‐flight into a parameter‐level, crosstalk‐immune strain metric without algorithmic overhead. A detailed comparative summary across reported soft TDR platforms—highlighting the distinct physical architectures, signal processing strategies, and our unique capability for parameter‐level crosstalk decoupling—is provided in Note S2.

Although the design concept presented here may appear conceptually reminiscent of our previous work on LM conduit‐based strain sensing [41], the underlying physical mechanisms and system architectures are fundamentally different. Our previous approach relied on continuous frequency‐domain phase accumulation along a single‐conductor line operating in a two‐port transmission mode, which suffered from environmental parasitic coupling and required an external physical reference line for baseline compensation. In contrast, this work establishes a single‐ended time‐domain reflection paradigm implemented on a parallel twin‐conductor line; it eliminates far‐end receiver wiring, naturally decouples localized impedance perturbations along the temporal axis, and provides intrinsic physical‐layer electromagnetic shielding without auxiliary reference channels. A comprehensive technical and physical comparison between these two approaches is summarized in Note S3.

The sensor was fabricated by filling EGaIn into highly stretchable elastomeric conduits, which can be cut to the desired length (Figure 1E; Figure S5A) [50]. To prevent leakage, a mechanically clamped end‐sealing design was employed. Specifically, slightly oversized, polished copper electrodes featuring a necked (dumbbell‐shaped) section were inserted into both ends of the conduit [51], and radial compression was applied at the necked region through mechanical fastening to form a tight and robust seal. For the parallel‐wire configuration, two LM‐filled conduits were aligned side‐by‐side and bonded using a thin layer of latex solution, which served as an adhesive elastomer matrix to ensure stable mechanical integration (Figure S5B). This structure supports reliable transmission‐line behavior under stretch. The functionality of the device was confirmed by time‐domain measurements, which showed well‐defined incident and reflected signals under square‐wave excitation (Figure S6), consistent with transmission‐line operation. To illustrate this behavior, the sensor was subjected to localized transverse compression while monitoring the reflection time‐of‐flight (Figure 1F). The arrival time of the primary reflection remains unchanged, indicating that the time‐of‐flight is governed solely by the total propagation path. This observation supports the length‐encoded sensing mechanism, whereby deformation modes that do not modify the propagation path are intrinsically excluded from the readout. It should be noted that the pulse frequency of the signal was defaulted to 50 kHz for standard laboratory characterization. Importantly, the time‐domain spatial resolution is inherently governed by the high‐frequency spectral components embedded within the pulse rise time rather than the pulse frequency itself. Practically, the pulse frequency can be flexibly chosen across a broad operating window below 10 MHz—above which residual high‐order multiple reflections begin to superimpose onto subsequent cycles (Figure S7)—while lower pulse frequencies simply adjust the waveform update density without compromising propagation accuracy.

### 2.2 Length Encoding Strain Sensing Performance of the LM–TDR Sensor

**FIGURE 2:**
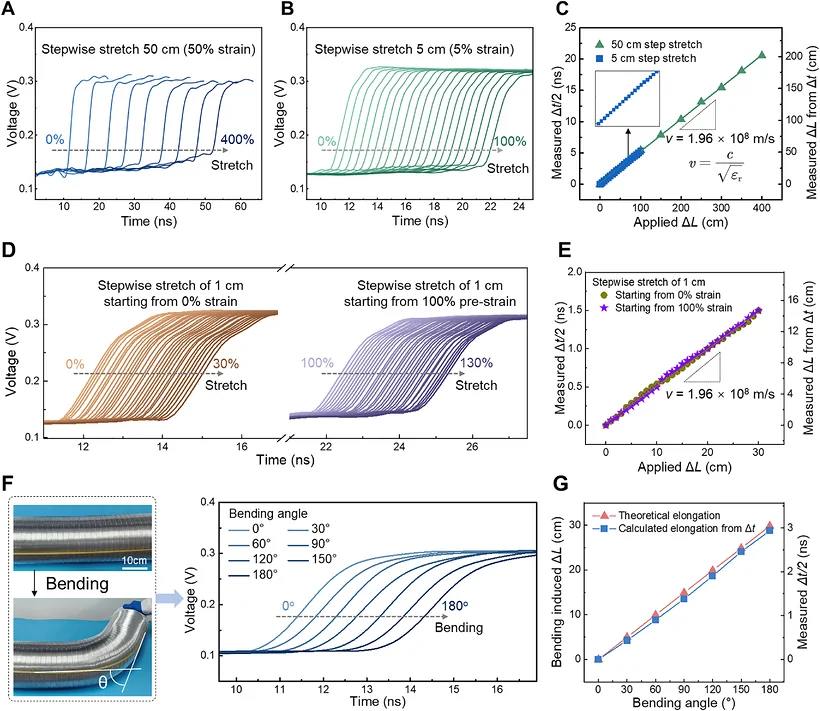
Wide dynamic range, strain‐independent resolution, and geometry‐encoded sensing. A‐C Wide working range. Reflected signals under stepwise stretching with large (50 cm, A) and moderate (5 cm, B) step sizes, and the corresponding relationship between applied elongation and measured elongation derived from reflection delay (C), demonstrating a linear response over a wide dynamic range. D‐E Strain range–independent resolution. Reflected signals under small stepwise stretching (10 mm) starting from the as‐fabricated state and from a pre‐strained reference state (D), together with measured vs. applied elongation (E), confirming strain‐independent spatial resolution. F‐G Global bending–induced elongation. Experimental configuration (F, left), reflected signals under varying bending angles *θ* (F, right), and comparison between theoretically predicted elongation and elongation extracted from reflection delay (G), validating that bending‐induced changes in propagation length are directly encoded in the time‐of‐flight readout.

Following validation of the propagation‐length–based sensing principle, the quantitative performance of the LM–TDR sensor was evaluated under tensile elongation. Sensor performance was first evaluated using a representative conduit with an initial length of 100 cm under stepwise tensile elongation (50 and 5 cm increments), during which the reflected voltage waveform exhibited progressive time‐of‐flight shifts proportional to the applied extension (Figure 2A). To eliminate subjective manual bias, an objective automated extraction workflow based on point‐by‐point differential peak detection of the averaged waveforms was implemented to precisely determine the round‐trip delay (Δ*t*), as indicated in Figure S8. The sensor supports a wide dynamic range, accommodating elongation up to 400% strain, confirming proportional scaling between delay and propagation path with a propagation speed of 1.96 × 10^8^ m/s (Figure 2A‐C).

Importantly, the sensor demonstrates a strain‐range‐independent axial length change resolution, reliably resolving a minimum elongation increment of 10 mm across the entire operating range (Figure 2D‐E). Under our current oscilloscope sampling rate (5 GSa/s, nominal sampling interval of 200 ps and propagation velocity of 1.96 × 10^8^ m/s), a single raw sampling point theoretically corresponds to a physical length discretization interval of 19.5 mm. By combining 16‐frame hardware waveform averaging with a windowed differential edge‐detection algorithm (Figure S8), sub‐sampling‐interval time delays are extracted, enabling an experimentally validated spatial displacement resolution of 10 mm—governed by the excitation edge rise time, system noise floor, and peak extraction algorithms. This 10 mm threshold defines the minimum detectable increment of global axial elongation rather than localized tactile spatial positioning in previous LM–TDR systems [3]. Crucially, this value defines the absolute spatial length resolution; the derived strain resolution inherently scales with baseline line length, meaning that longer sensors achieve progressively finer strain resolution. Unlike conventional flexible strain sensors, whose resolution often deteriorates at small deformation due to nonlinear electromechanical coupling [7, 52, 53, 54, 55], the LM–TDR readout preserves consistent precision because the time‐of‐flight directly reflects absolute propagation length [56]. While this precision effectively captures macro‐scale global deformations, finer spatial resolution can be systematically achieved in future designs through hardware and structural optimizations—such as adopting higher sampling rates, refined sub‐sample edge‐fitting algorithms, or incorporating serpentine conduit geometries that amplify the local propagation path per unit deformation.

Although stretching reduces the conduit diameter, such variations have negligible influence on the propagation velocity, preserving the length‐based encoding mechanism. Conduits with different diameters but identical lengths exhibit identical responses (Figure S9), and consistent propagation velocities are observed across sensors with different initial lengths (Figure S10). These indicate that the propagation velocity is governed primarily by the effective dielectric environment rather than conductor geometry. Notably, this mechanism is compatible with different elastomeric materials, as sensors fabricated with silicone tube and Ecoflex 00–30 exhibit comparable responses (Figure S11).

In addition to uniaxial stretching, the sensor captures geometric elongation arising from global bending, which increases the propagation path length along the outer surface of a structure. When a substrate is bent, the outer surface undergoes elongation proportional to the bending angle, leading to a predictable increase in propagation distance (Figure S12). To validate this effect, an LM conduit was conformally attached to a cylindrical substrate and subjected to controlled bending (Figure 2F‐G). As the bending angle increased, the reflection time‐of‐flight exhibited systematic shifts that closely matched the geometrically predicted arc‐length change. These results confirm that the sensing output is determined by propagation path length, regardless of deformation mode. Conversely, when the sensor undergoes free‐body bending, spatial routing, or flexure along its structural neutral axis without changing its net physical path length, the round‐trip propagation time remains virtually invariant (Figure S12B). This intrinsic immunity to non‐elongative configural changes enables conformal integration across complex geometries without introducing false strain readings (see the discussion below).

### 2.3 Robust Strain Sensing Under Multimodal Perturbations of the LM–TDR Sensor

**FIGURE 3:**
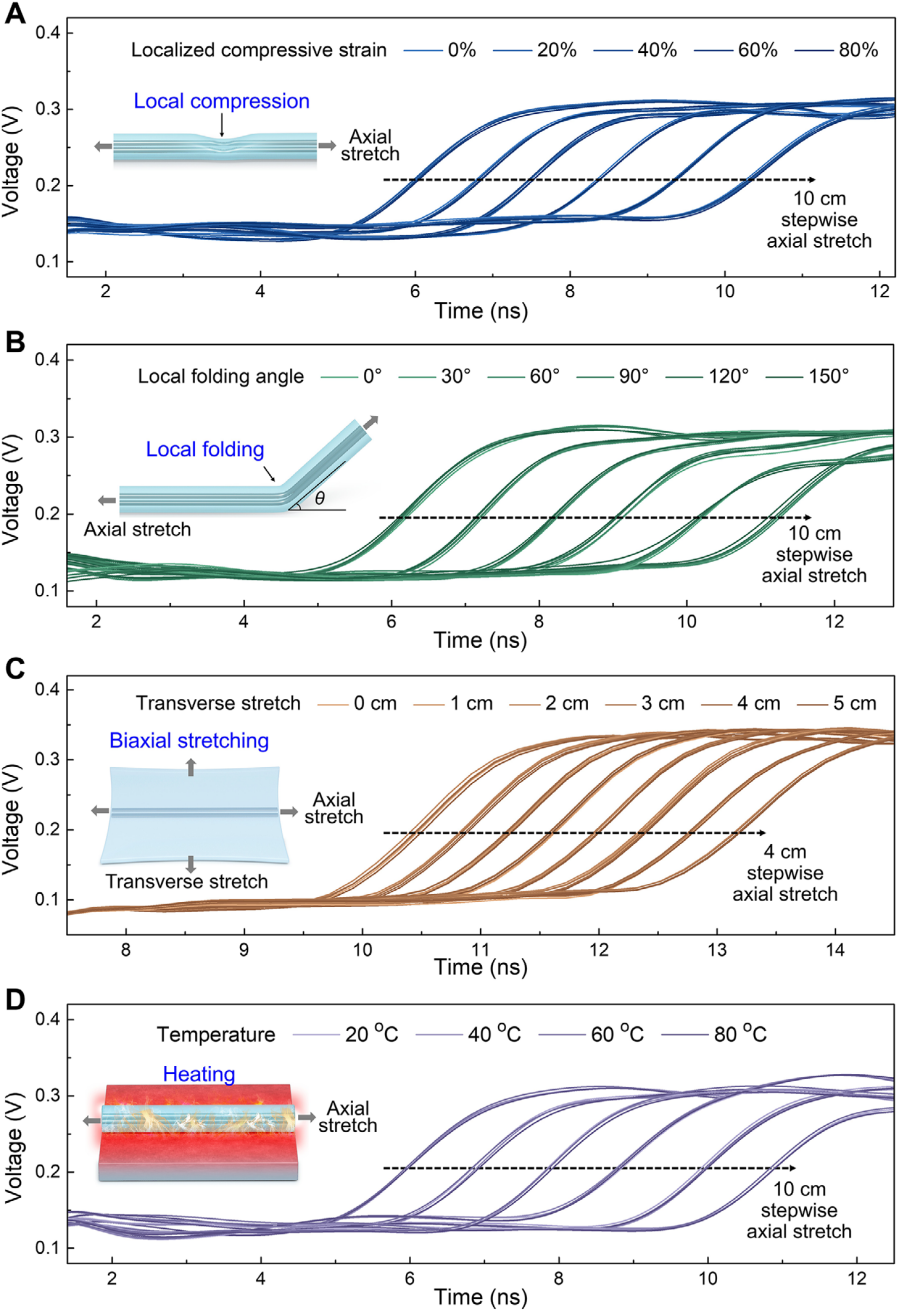
Robustness of the LM–TDR sensor against off‐axis deformation and thermal perturbation. A Response during stepwise axial stretching (10 cm increments) under simultaneous localized transverse compression (0%–80%). B Reflected waveforms during stepwise axial stretching (10 cm increments) under localized folding over a wide bending angle (0°–150°). C Biaxial stretching with axial elongation and simultaneous transverse stretch. D Temperature response from 20°C–80°C under stepwise axial stretching (10 cm increments). Across all conditions, the reflection time‐of‐flight is governed solely by axial elongation, demonstrating intrinsic insensitivity to transverse deformation and thermal perturbations.

A key advantage of the LM–TDR sensor is its intrinsic selectivity to axial strain, even under substantial multimodal deformation (Figure 3). To examine this behavior, an LM conduit was progressively elongated in 10 cm steps while localized transverse perturbations were applied at its midpoint at different amplitudes. Local compressive strains of 0%–80% produced no measurable change in the arrival time of the primary reflection, indicating that transverse compression does not affect total propagation length determination (Figure 3A; Figure S13A). Similarly, localized folding over a wide angular range (0–150°)—applied locally at the conduit without altering its total axial path length—did not alter the primary reflection from the open end (Figure 3B; Figure S13B). Even when multiple compressions or folds were simultaneously introduced at different positions, the time‐of‐flight remained governed solely by the total axial length (Figure S14). These results demonstrate that the sensing output depends exclusively on global axial elongation, while local off‐axis perturbations do not contribute to the measured signal. It should be noted that when compression or folding becomes sufficiently severe to rupture the LM conductor and form a new open circuit, the reflection position shifts accordingly (Figure S15). In this case, the effective propagation length is physically redefined by the newly formed electrical discontinuity.

This geometric length‐encoding principle further extends to biaxial stretching, a scenario that remains challenging for amplitude‐based strain sensors [4, 16, 27, 55]. Because the time‐of‐flight is governed solely by the propagation path along the sensing axis, deformation components orthogonal to this axis are intrinsically rejected (Figure 3C). Consequently, orthogonal or non‐uniform transverse deformations produce localized impedance variations and reflection amplitude changes, but introduce little phase‐velocity alteration or cumulative time‐delay error (Note S4). To demonstrate this capability, the LM conduits were encapsulated in Ecoflex 00–30 to permit simultaneous axial and transverse stretching. Under uniform biaxial loading (stepwise axial and transverse stretching of 40 and 10 mm, respectively), the time‐of‐flight responded exclusively to axial deformation, while transverse strain produced no measurable influence on the readout (Figure 3C; Figure S16A). Furthermore, when localized non‐uniform transverse stretches up to 40 mm were applied across the central region while holding the conduit at distinct step‐wise axial elongation states, the TDR reflection waveforms maintained invariant terminal time delays with negligible deviation (2.1%) at each given axial length. This confirms that intermediate impedance discontinuities do not degrade terminal reflection detection (Figure S16B‐C). Note that, despite occurrences of LM breakage and the formation of new insulative oxide layers during deformation, their ultrathin nature (0.5–3 nm) ensures minimal impact on the conductive path [57, 58]. Altogether, these theoretical and experimental results verify the intrinsic axial selectivity and robust length‐sensing fidelity under complex multiaxial states.

Beyond mechanical perturbations, temperature fluctuations also challenge conventional amplitude‐based sensors, because amplitude‐based transduction mechanisms are intrinsically temperature sensitive [7, 59, 60, 61]. While structural or material designs that leverage mismatches between thermal and mechanical responses can mitigate this cross‐sensitivity, they typically do not fully decouple temperature and strain in the readout. In contrast, the LM–TDR sensor exhibits robust performance over 20°C–80°C (Figure 3D). An LM conduit was subjected to axial elongation while being heated (Figure S17). Despite temperature variation, the reflection time‐of‐flight showed negligible change (Figure 3D; Figure S13C). This stability arises from the nearly temperature‐invariant propagation velocity governed by the effective dielectric constant of the elastomer. For natural rubber used in this study, the effective dielectric response varies weakly over 20°C–80°C, and this range lies far above its glass‐transition temperature, so the conduit remains in a stable rubbery regime without dielectric anomalies associated with phase transitions [62].

Although LMs possess unique liquid mobility and thermal stability, physical scratches and leakage remain a common, intrinsic challenge for stretchable conduits. To examine these operational boundaries, we systematically evaluated the sensor's response under localized physical damage (Note S5). A distinct advantage of our TDR‐based platform is that the specific damage state can be directly identified and diagnosed from the reflection waveform morphology. Minor surface scratches that preserve internal LM continuity introduce only weak, localized impedance mismatches without degrading the primary terminal echo extraction (Figure S18A). Conversely, severe damage causing complete channel interruption (open‐circuit) or twin‐conductor short‐circuiting induces total reflection or phase inversion at the scratch site, causing the primary echo to shift or vanish (Figure S18B‐C). Consequently, under extreme damage, the TDR system gracefully transitions from full‐length strain tracking to precise spatial damage localization along the soft conduit, providing predictable self‐diagnostic capabilities unachievable by conventional flexible strain sensors.

### 2.4 Intrinsic Self‐Referenced and Calibration‐Free Strain Sensing of the LM–TDR Sensor

**FIGURE 4:**
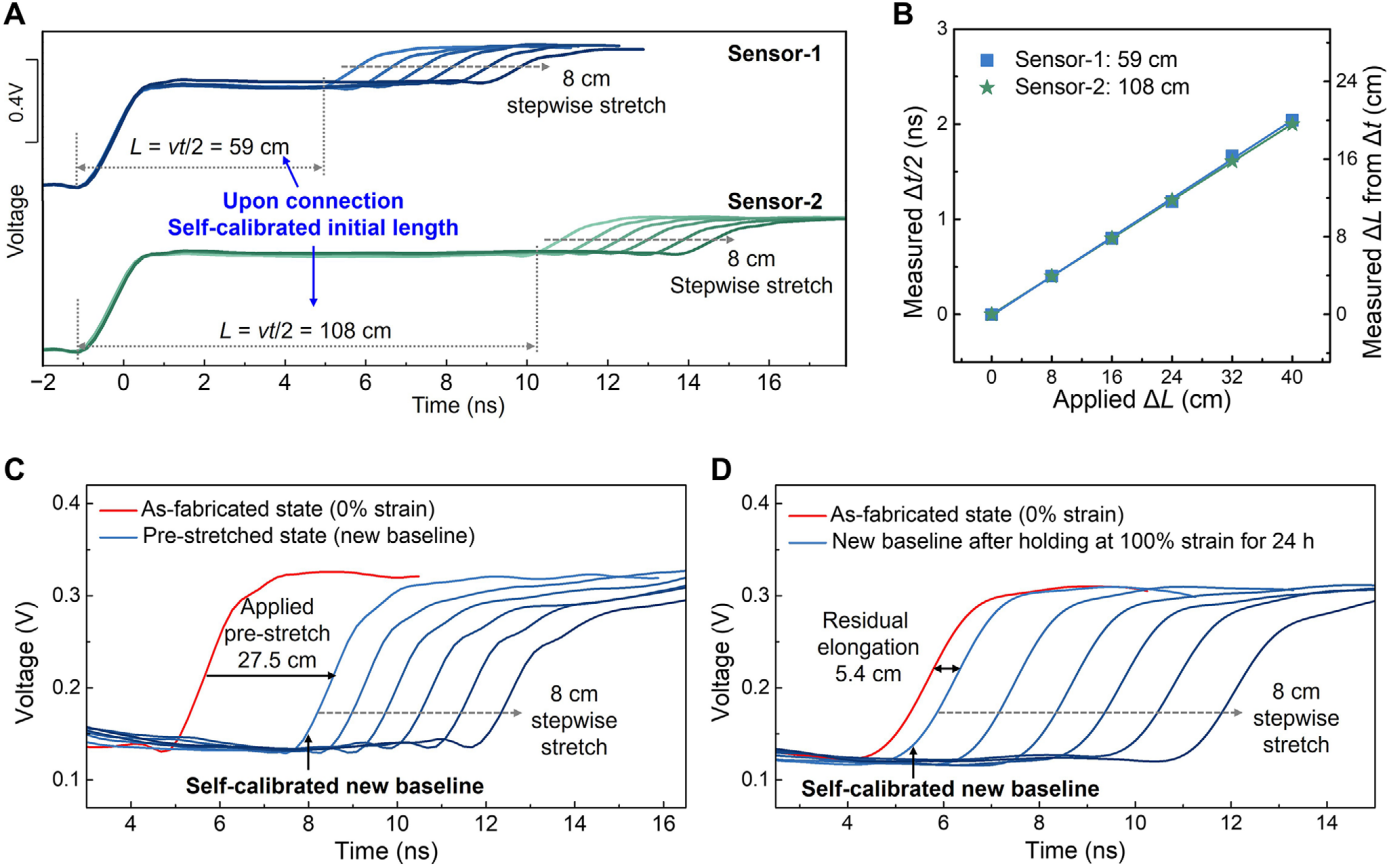
Inter‐sensor consistency and flexible baseline definition enabled by self‐calibration. A‐B Inter‐sensor consistency. Reflection waveforms from two independently fabricated LM–TDR sensors (Sensors 1 and 2) under stepwise stretching (8 cm increments) show systematic shifts with increasing elongation (A). The measured elongation and reflection‐delay change vs. applied elongation exhibit consistent, linear, and calibration‐free responses across sensors with different self‐calibrated initial lengths (B). C Self‐calibrated baseline definition for deformation history. The length readout enables the reference state to be defined at the time of use; a pre‐stretched state can be adopted as a new baseline for subsequent measurements. D Self‐calibrated baseline definition for durability. Accurate elongation measurement is maintained after irreversible deformation induced by prolonged holding, by redefining the baseline following such conditioning histories.

By encoding deformation directly into absolute propagation length, the LM–TDR sensor provides a geometry‐defined, self‐referenced readout. Because the sensor directly measures its physical length in real time, the reference state is inherently defined by the device geometry itself. This self‐calibrated nature yields three practical advantages for deployment and long‐term operation (Figure 4).

First, this mechanism ensures inter‐sensor consistency and calibration‐free deployment. Upon connection, the absolute length of each sensor is directly obtained from its reflection, serving as an intrinsic reference (Figure S19). Independently fabricated devices can therefore be interpreted on a common physical scale without matching material properties or performing individual calibration, as demonstrated using two LM–TDR sensors (Sensors 1 and 2) which are subsequently used for precise elongation measurement without further calibration (Figure 4A‐B).

Second, any post‐installation deformation can be defined as a new reference state. Because the readout depends only on the absolute length at the time of use, pre‐strain and mounting‐induced deformation do not compromise subsequent measurements. For example, a 59 cm conduit elongated to 86.5 cm was defined as the reference state, from which additional stretch was directly measured (Figure 4C; Figure S20).

Third, the sensor maintains measurement fidelity under complex mechanical histories and progressive irreversible deformation. During 10 continuous loading–unloading cycles at representative strains of 50%, 100%, and 200%, the sensor consistently tracked both the peak target displacements (maximum measurement error < 4.6%) and the progressive viscoelastic permanent set introduced after each relaxation (Figure S21). Independent benchmarking with a high‐precision digital caliper confirmed that the absolute lengths derived from TDR time delays matched physical measurements with negligible deviation, verifying that zero‐point redefinition introduces no cumulative error or sensitivity drift (Figure S21D). Rather than causing measurement failure, our TDR sensor continuously and accurately tracked the exact residual length increment of the conduit upon relaxation (Figure S21E). Even after severe mechanical history—including prolonged holding at 100% strain for 24 h and 200 cycles at 100% strain, which induced substantial permanent set of 5.4 cm and 3.4 cm, respectively—the sensor inherently retained its absolute displacement sensitivity and continued to report precise deformation without recalibration (Figure 4D; Figure S22).

Furthermore, this intrinsic reference scheme together with the single‐ended TDR operation enables highly versatile installation architectures. For compact target regions under tight spatial constraints (e.g., 1–5 cm localized length tracking), the soft conduit can be seamlessly routed such that only the designated 1–5 cm terminal segment is bonded to the high‐strain area, while the upstream portion functions as an integrated interconnect line (Note S6). These results demonstrate that the LM–TDR sensor operates as a self‐referenced system, enabling reliable strain measurement independent of device variation, installation condition, and mechanical history.

### 2.5 The Practicability of the LM–TDR Sensor

**FIGURE 5:**
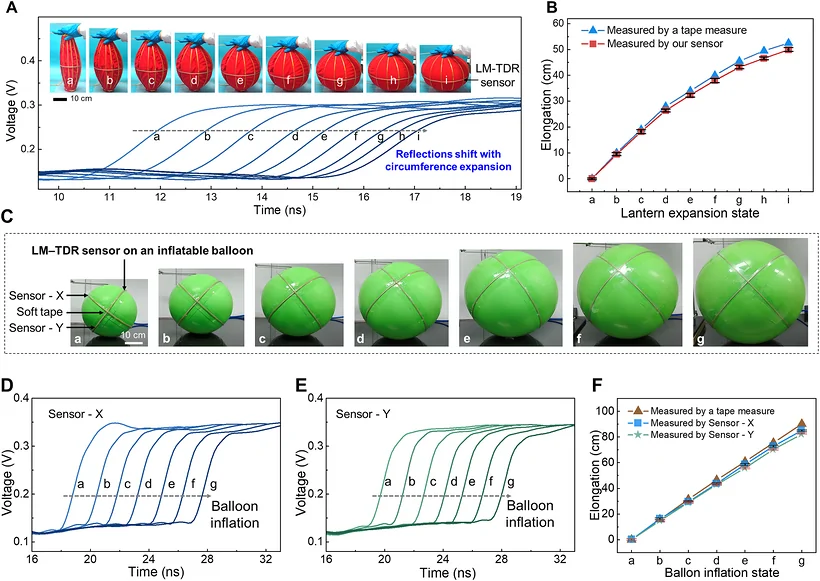
Practical applicability under geometric complexity and intrinsic multidirectional strain selectivity. A‐B Length tracking on a geometrically complex surface. An LM–TDR sensor integrated with a foldable lantern experiences distributed deformation from internal ribs and expansion‐induced elongation (A). The sensor accurately tracks global length change, in agreement with reference measurements (B). C Multiaxial sensing configuration. Two LM–TDR conduits arranged orthogonally on an inflatable membrane enable independent strain readout along two directions during equiaxial inflation. D–E Direction‐selective response. Reflected waveforms from sensors aligned along the X‐ and Y‐directions show that each channel responds exclusively to elongation along its own axis during inflation. F Quantitative comparison of elongation measured along orthogonal directions and by a tape‐measure reference, demonstrating accurate and decoupled multiaxial strain sensing. The error bars in (B) and (F) represent standard deviations obtained from three measurements using same samples.

**FIGURE 6:**
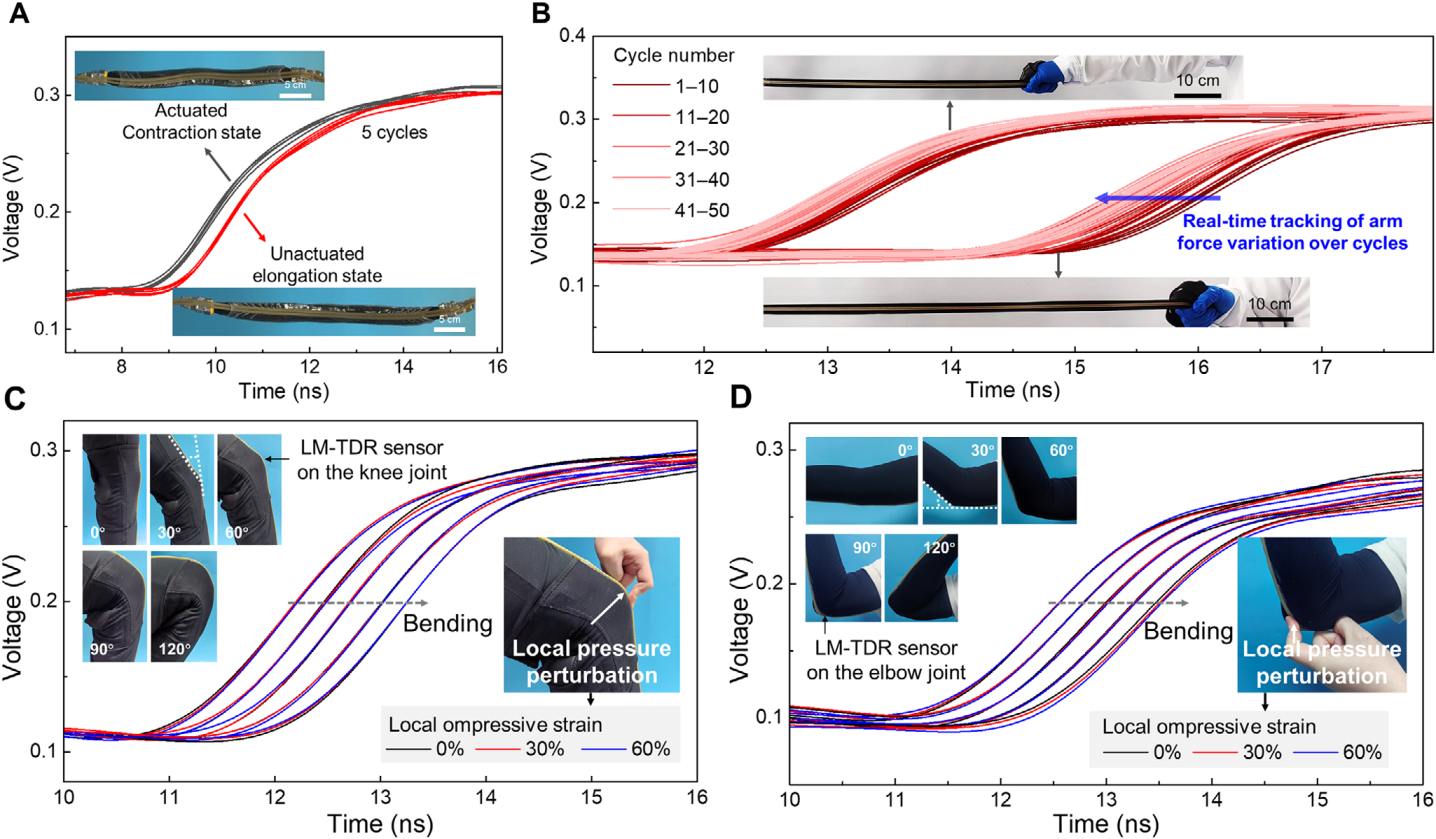
Robust operation under sustained pre‐strain, dynamic loading, and wearable perturbations. A Operation under sustained pre‐strain. A pneumatic artificial muscle is monitored by a pre‐stretched sensor, which continuously tracks contraction and relaxation over 5 cycles. B Dynamic loading under pre‐strain. The sensor tracks band elongation during repetitive stretching by arm, with cycle‐to‐cycle variations in peak elongation reflecting changes in applied force, demonstrating robust sensing under dynamic loading and transient perturbations. C–D Wearable robustness under external perturbations. LM–TDR sensors integrated across knee (C) and elbow (D) joints track bending‐induced elongation over varying joint angles. Despite intentional localized pressing and transverse compression, the reflection waveforms shift consistently with joint bending, indicating that the time‐of‐flight is governed solely by axial deformation and is insensitive to external disturbances.

**FIGURE 7:**
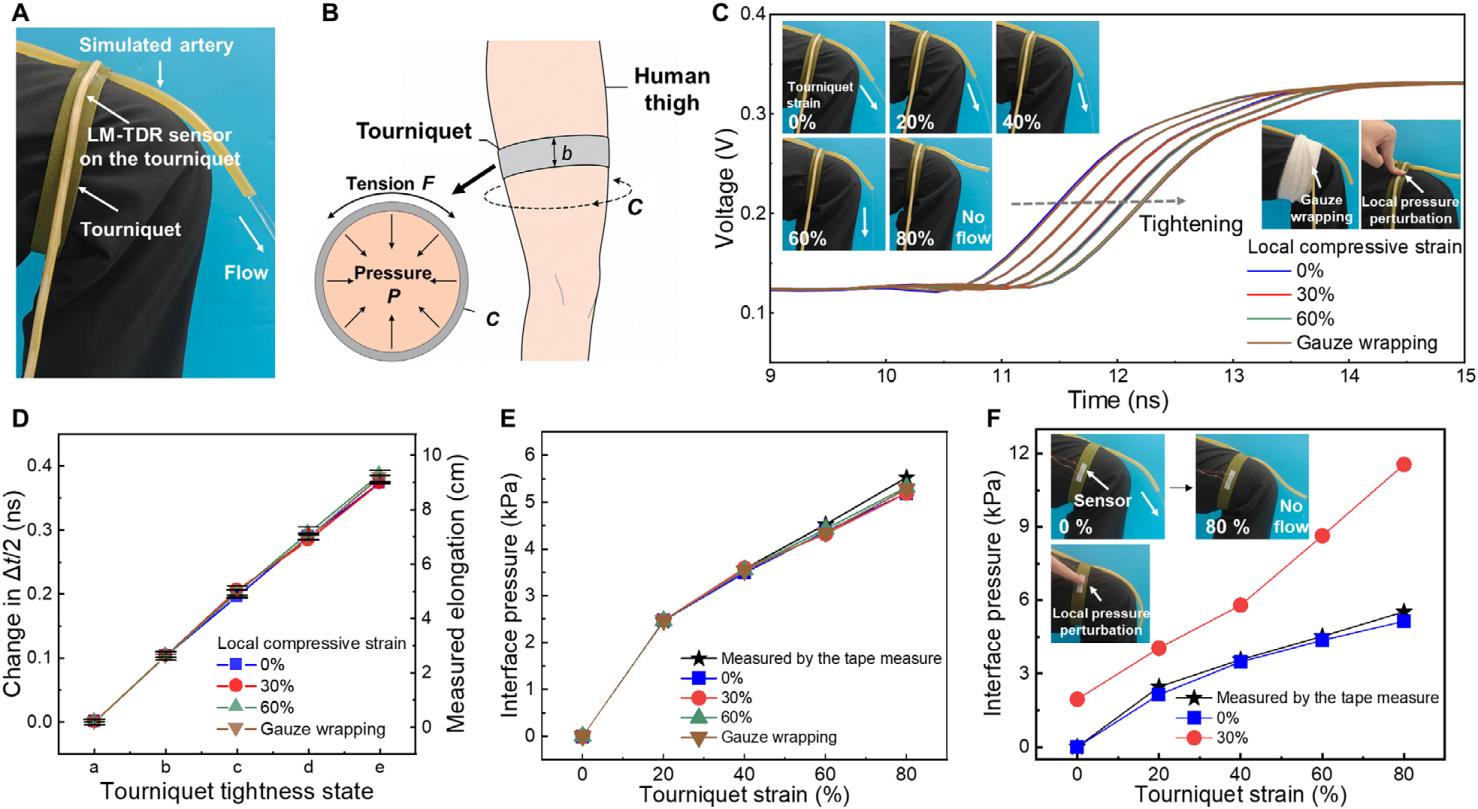
Task‐level tourniquet tightness monitoring for safe and effective hemostasis. A Clinical scenario and task of tourniquet tightness monitoring using the LM–TDR sensor. A simulated artery was used to evaluate flow occlusion during tightening. B Schematic illustration of tourniquet pressure calculation, where *F* is the circumferential tension, *b* is the tourniquet width, and *C* is the thigh circumference. C Reflected waveforms during progressive tourniquet tightening and under external perturbations. D Time‐delay changes and corresponding measured elongations at different tourniquet tightness states under different local compressive conditions. E Interface pressure derived from the LM–TDR sensor under different tourniquet strains, showing close agreement with the values measured by the tape measure despite local compression and gauze wrapping. F Comparison with a conventional flexible strain sensor under local pressure perturbation, showing substantial deviation from the tape‐measure reference under 30% local compression. The error bars in (D) and (E) represent standard deviations obtained from three measurements using same sensors.

To evaluate the practical applicability of the LM–TDR sensor under realistic operating conditions, we investigated its performance across three progressive operational dimensions: spatial multiaxial geometry, dynamic biomechanical tracking, and task‐level clinical intervention (Figures 5, 6, 7). Note that, in all demonstrations, the sensor was operated directly from its installed state, without eliminating pre‐curvature, pre‐strain, or performing post‐installation recalibration. During the experiment, the tourniquet was progressively tightened under quasi‐static step‐wise conditions—holding the deformation state fixed at each milestone to capture the precise TDR waveform (achieved with a high‐speed single‐waveform acquisition time of 0.32 ms) before advancing to the next state, ensuring uncompromised measurement accuracy under each complex physical state. Serving as proof‐of‐concept demonstrations rather than fully deployed systems, these scenarios showcase the sensor's versatile capabilities across a diverse range of real‐world applications—spanning from monitoring shape and volumetric deformation in deployable structures (e.g., parachutes and hot air balloons) to dynamic human motion tracking and reliable clinical interventions. To rigorously quantify measurement accuracy, the absolute lengths extracted by the TDR system were benchmarked against physical lengths measured using a standard measuring tape across various operating ranges, yielding a maximum relative error of less than 8.8% (Figure S26).

We first examined the sensor's response under geometrically complex topologies and multiaxial spatial deformations (Figure 5). For conformal length tracking, an LM conduit was attached to a foldable lantern structure (Figure 5A), where it experienced distributed deformation from internal supporting ribs and incidental expansion‐induced elongation (Figure S27). Despite these geometric complexities and multi‐point disturbances, the sensor accurately tracked the global length change, confirming reliable operation on curved and irregular surfaces (Figure 5B). To further extend this capability into multi‐dimensional spatial mapping, two LM–TDR conduits were arranged orthogonally on a deformable inflatable membrane (Figure 5C; Figure S28), which were operated simultaneously to provide independent strain readouts along each axis during equiaxial stretching. During equiaxial expansion, each channel responded exclusively to path‐aligned elongation while remaining completely immune to orthogonal deformation (Figures 5D‐F). This behavior arises from the propagation‐based sensing mechanism, in which only path‐aligned deformation contributes to the time‐of‐flight, enabling scalable multiaxial strain mapping on deformable structures without complex signal decoupling algorithms. Furthermore, to complement this mechanical decoupling, we verified that signal propagation along one line induces little electromagnetic interference in the adjacent channel during simultaneous acquisition (Note S7). Because the two conduits intersect orthogonally (*E*_A_⊥*E*_B_), mutual coupling is physically confined to a discrete crossover point. Experimental results confirm that orthogonal channels exhibit negligible crosstalk even at direct contact, ensuring robust, multi‐axis strain tracking without signal degradation (Figure S29).

Next, we evaluated performance under high sustained pre‐strain, dynamic loading, and human joint motion (Figure 6). In pneumatic artificial muscles requiring large initial stretch (Figure S30), the sensor was pre‐stretched beyond the actuator's maximum contraction after installed, accurately tracking instantaneous muscle length across repeated pressurization cycles without baseline drift (Figure 6A). Similarly, during exercise using elastic resistance bands (Figure 6B; Figure S31), the TDR readout continuously captured peak elongations, with cycle‐to‐cycle variations reflecting changes in arm force, in quantitative agreement with a commercial force gauge. When integrated into wearable sleeves spanning human elbow and knee joints (Figure 6C‐D), the sensor precisely tracked joint flexion dynamics. Remarkably, even when intentional localized compression or external touching was applied during joint motion, the time‐of‐flight responded strictly to axial joint bending without signal artifacts, proving drift‐free stability under complex biomechanical conditions.

In emergency first‐aid and limb surgeries, precise tourniquet pressure control is vital—insufficient compression fails to arrest lethal hemorrhage, while excessive pressure risks irreversible nerve damage and tissue necrosis [63] (Figure S32). During practical application around a human limb, the sensor encounters severe multimodal mechanical interference, including simultaneous circumferential bending around complex anatomical contours, direct transverse pressing from the elastic band, soft‐tissue compliance, surgical gauze wrapping, and accidental tactile touching. Under such complex conditions, conventional strain sensors suffer from severe signal crosstalk and catastrophic measurement artifacts. To overcome this fundamental challenge and establish task‐level real‐world utility, we integrated the LM–TDR conduit onto a commercial elastic tourniquet for robust, real‐time hemodynamic occlusion pressure monitoring (Figure 7). To quantitatively evaluate its performance, we established a hybrid hemodynamic setup, where a fluid‐perfused elastic conduit was conformally mounted onto the surface of a human thigh to simulate a superficial pulsing artery (Figure 7A). Because the TDR readout mechanism directly tracks the absolute electromagnetic propagation distance, it accurately extracts the pure axial strain (*ε* = Δ*L*/*L*_0_) of the tourniquet. By mapping this measured strain onto the intrinsic force‐strain profile of the elastic band (Figure S33), the applied axial tensile force (*F*) is continuously quantified. Specifically, according to the generalized Law of Laplace *p* = 2π*F*/(*bC*), the radial compression pressure (*P*) applied to the limb scales directly with this axial tensile force (*F*), as shown in Figure 7B.

During the experiment, the tourniquet was progressively tightened. Specifically, the LM–TDR sensor was conformally attached to a 10‐cm‐long active region of the elastic tourniquet to ensure synchronous deformation. A flexible ruler was utilized to precisely measure the length changes of the sensor‐integrated region during each tightening step, and the corresponding fluid flow states and TDR waveforms were recorded simultaneously. As shown in Figure 7C–E, the TDR system continuously captured these step‐wise strain increases and translated them into real‐time radial pressure dynamics, successfully quantifying the actual compression pressure exerted on the thigh. Consequently, as the axial tension increased, the fluid flow rate within the simulated artery diminished systematically (Figure 7C, inset), achieving complete hemodynamic occlusion (zero flow rate) precisely at an axial strain of 80%, where the extracted radial pressure reached approximately 6 kPa. While this surface pressure is lower than the typical clinical occlusion threshold (30–40 kPa) required for human limbs, this discrepancy is primarily attributed to soft‐tissue compliance and the anatomical depth of real arteries beneath thick muscle layers, whereas our hybrid setup models a superficial vessel [63]. Nevertheless, the system reliably tracks the continuous pressure dynamics. Notably, throughout this entire tightening process, the LM–TDR sensor maintained impeccable accuracy, completely unaffected by the intense external compression and multi‐layered surgical gauze wrapping (Figure 7C‐E). In stark contrast, when subjected to these identical real‐world perturbations, conventional piezoresistive strain sensors failed catastrophically, exhibiting severe false strain spikes and signal drifts (Figure 7F), thereby confirming the absolute reliability of our TDR architecture for complex clinical interventions.

## 3 Conclusion

We demonstrate a stretchable LM–TDR strain sensor that determines deformation by directly measuring the time‐of‐flight of an electromagnetic pulse along a LM transmission line. Because the readout depends solely on the total propagation length, the sensor operates intrinsically from its installed configuration without baseline calibration and remains insensitive to off‐axis mechanical perturbations, including compression, bending, transverse stretching, and temperature variation. The sensor exhibits a linear response over a wide working range up to 400% strain, together with a strain‐range–independent spatial resolution of 10 mm. Its performance is largely independent of conduit geometry and material choice, showing consistent propagation behavior across different diameters, lengths, and elastomeric substrates. In addition, enabled by its self‐referenced operation, the device maintains accurate strain readout under large pre‐strain, cyclic loading, and irreversible deformation, highlighting its robustness under realistic mechanical conditions.

To validate its practical applicability, we further demonstrate reliable strain sensing on geometrically complex surfaces, in pneumatic artificial muscle systems under sustained pre‐strain, during dynamic loading driven by human motion, in multiaxial deformation configurations, and through task‐level clinical tourniquet monitoring for real‐time hemodynamic occlusion pressure control. These results confirm accurate and repeatable global length tracking under mechanically complex and multidirectional conditions, while highlighting exceptional resilience against severe real‐world mechanical interferences such as transverse compression and tissue compliance. This work establishes length‐defined time‐of‐flight measurement as a practical route to intrinsic parameter‐level decoupling, enabling strain sensing without signal reconstruction or scenario‐dependent calibration. These findings provide a foundation for robust strain sensing in wearable electronics, soft robotics, translational medical interventions, and deformable structural systems [64, 65, 66, 67, 68].

While straight parallel twin‐lines are utilized here to demonstrate the fundamental sensing physics, compact serpentine geometries can be further adopted to amplify total delay within a constrained footprint. However, such non‐linear layouts must maintain an adequate spacing between adjacent parallel lines to avoid mutual inductive/capacitive crosstalk and waveform degradation, serving as a valuable avenue for high‐density sensor integration in future work. Furthermore, in this study, benchtop instruments were utilized to ensure precise laboratory‐grade data acquisition for validating the underlying sensing physics. In modern electronics, TDR signal generation, sampling, and time‐delay extraction do not inherently rely on bulky hardware, as portable implementations have been broadly commercialized (e.g., handheld cable testers and compact TDR modules, summarized in Table S3). Nevertheless, comprehensive characterizations of high‐frequency dynamic metrics—such as precise step‐response times, mechanical hysteresis, and ultra‐fast motion tracking—were constrained in our current setup due to frame‐averaging delays and data‐transfer protocols of general‐purpose oscilloscopes, which hinder rapid, high‐throughput waveform archiving. Future integration with custom ASICs, compact FPGA modules, or dedicated high‐speed TDR chipsets will overcome current instrumentation bottlenecks, unlocking fully portable, high‐bandwidth wearable TDR sensing systems for soft robotics and personalized healthcare.

## Author Contributions

**Zhang Liu**: methodology, investigation, formal analysis, writing – original draft. **Fengdeng Jin**: methodology, formal analysis, investigation, writing – original draft. **Wenxuan Shi**: writing – original draft, investigation, formal analysis, visualization. **Meng She**: conceptualization, writing – original draft, writing – review and editing, investigation. **Ziqi Meng**: investigation, formal analysis. **Miao Fang**: investigation, formal analysis. **Yixin Zhang**: investigation, formal analysis. **Qianwen Dong**: investigation, formal analysis, visualization. **Wandi Yang**: investigation, formal analysis, visualization. **Lei Shi**: funding acquisition, supervision, writing – review and editing. **Xujia Zhao**: investigation, formal analysis. **Feng Zhou**: conceptualization, supervision, writing – review and editing, funding acquisition. **Bin Yao**: conceptualization, investigation, supervision, funding acquisition, writing – review and editing, writing – original draft.

## Conflicts of Interest

The authors declare no conflicts of interest.

## Supporting information




**Supporting File**: advs77839‐sup‐0001‐SuppMat.docx.

## Data Availability

The data that support the findings of this study are available from the corresponding author upon reasonable request.
