# Supplementary material for: Geometry‐Encoded Soft Strain Sensing via Liquid‐Metal Transmission Lines
Source: Adv Sci (Weinh). 2026 Sep 16:e77839. Online ahead of print. doi: 10.1002/advs.77839 (PMC13583088; doi:10.1002/advs.77839)
Supplement: Supplementary file 1 [file ADVS-9999-e77839-s001.docx]

**Supplementary information**

**Geometry-Encoded Soft Strain Sensing via Liquid-Metal Transmission Lines**

*Zhang Liu, Fengdeng Jin,* *Wenxuan Shi, Meng She*, Ziqi Meng, Miao Fang,* *Yixin Zhang,* *Qianwen Dong, Wandi Yang, Lei Shi, Xujia Zhao, Feng Zhou, Bin Yao**

Z. Liu, F. Jin, W. Shi, Y. Zhang, L. Shi, F. Zhou, B. Yao

School of Aerospace Science and Technology

Xidian University

Xi’an 710071, China

M. She

Honghui Hospital

Xi’an Jiaotong University

Xi’an, 710000, China

Z. Meng, X. Zhao

School of Electronic Engineering

Xidian University

Xi’an 710071, China

M. Fang, W. Yang

School of Mechano-Electronic Engineering

Xidian University

Xi’an 710071, China

Q. Dong

School of Optoelectronic Engineering

Xidian University

Xi’an 710071, China

Zhang Liu, Fengdeng Jin, and Wenxuan Shi contribute equally to this work.

E-mail: shemengsdu@gmail.com (M. S.); binyao@xidian.edu.cn (B.Y.)

**Materials and methods**

**Materials**: Eutectic gallium–indium (EGaIn, melting point of 16 °C) was purchased from Dongguan Dingguan Metal Technology Co., Ltd. (China) and used as received. Latex elastomer tubing and natural latex were obtained from Juyuan Polymer Co. Ecoflex 00-30 and silicone tubing were purchased from Smooth-On. Ecoflex 00-30 silicone elastomer was prepared by mixing Part A and Part B at a 1:1 mass ratio. Natural latex adhesive was prepared by mixing latex with curing agent at a mass ratio of 20:3.

**Sensor fabrication**: Latex tubes were cut to the desired length according to the intended application. Two tubes were aligned in parallel and brought into close contact. The prepared natural latex adhesive was uniformly applied along the interface between the two tubes and allowed to cure at room temperature for 48 h. After self-bonding, the two tubes formed an integrated structure, ensuring mechanical integrity under complex deformation while maintaining stable transmission-line characteristics. Liquid metal (LM) was injected into each tube using a syringe to ensure continuous filling without air bubbles. Copper wires were inserted into both ends to facilitate electrical connection to the measurement system. The tube ends were mechanically sealed to prevent LM leakage.

For sensors fabricated using Ecoflex 00-30, a casting and demolding method was employed. First, mixed Ecoflex was poured into a PTFE square mold and cured in an oven at 80 °C for 120 min to form a base substrate. Spray-coated rigid cylindrical rods (used as removable molds) were then placed in parallel on the substrate. Additional Ecoflex was poured to fully encapsulate the rods, followed by thermal curing at 80 °C. After curing, the cylindrical rods were carefully withdrawn from the edges to form hollow microchannels. LM was injected into the channels using a syringe, and copper wires were inserted at one end for electrical connection. The wired end was subsequently encapsulated with additional Ecoflex to ensure robust sealing.

**Electrical characterization**: A 50 kHz square-wave signal was generated using a signal generator as the excitation source. Reflected signals were recorded using a Tektronix DPO 4104B-L oscilloscope with a sampling rate of 5 GSa s⁻¹ (corresponding to a temporal resolution of 200 ps). A T-type connector was used to connect the signal generator, oscilloscope, and LM–TDR sensor simultaneously. The oscilloscope recorded the superimposed incident and reflected waveforms. The time delay (*t*) between the rising edge of the incident pulse and the reflected pulse was determined from the waveform. The instantaneous sensor length was calculated by, *L* = *vt*/2, where $v$ is the signal propagation velocity.

**Demonstration**: For monitoring human motion (*e.g.*, knee and elbow bending), the LM–TDR conduits were attached to selected positions on wearable fabrics, and waveform signals were recorded during motion. For deformation measurements on elastic or deformable structures, including inflatable balloons, collapsible lanterns, pneumatic artificial muscles, and resistance bands, the sensors were securely fixed using elastic adhesive tape (3M VHB 4905) to ensure stable attachment and accurate data acquisition under dynamic loading. For the task-level clinical tourniquet evaluation, the LM–TDR sensor was conformally integrated onto a 10-cm-long active region of a commercial elastic tourniquet, which was wrapped around a hybrid human-thigh hemodynamic setup incorporating a fluid-perfused elastic conduit. During progressive tightening in 2 cm elongation increments of LM conduit, length variations were monitored using a flexible ruler, and fluid flow dynamics were simultaneously recorded under severe mechanical interferences such as multi-layered surgical gauze wrapping and external manual compression.

Note that across all demonstration experiments, although the TDR waveforms were captured using a high-speed acquisition protocol—driven by a 50 kHz interrogation frequency (corresponding to a 20 µs period) and combined with a 16-frame waveform averaging protocol for noise reduction, yielding a single-dataset acquisition time of only 0.32 ms—the evaluations were conducted under quasi-static, step-wise conditions.

**Figure S1** **Equivalent distributed circuit model of the LM transmission line.** When the length of a transmission line becomes comparable to the operating wavelength, Kirchhoff laws are no longer directly applicable. In this case, the transmission line can be divided into infinitesimal segments, each much shorter than the wavelength, such that Kirchhoff laws can be applied locally to each segment, leading to the equivalent circuit model. Due to the finite conductivity of the conductors, resistive losses can be represented by a series resistance. The magnetic field induced around the conductors gives rise to inductance, which is modeled as a series inductance. The electric field between the two conductors results in distributed capacitance, represented by a shunt capacitance. If the surrounding medium is non-ideal, dielectric losses are present and can be modeled as a shunt conductance. The entire transmission line can thus be regarded as a cascade of these equivalent circuit elements.

**Supporting Information Note S1: Environmental stability comparison of parallel two-conductor and single-conductor line**

To establish the physical foundation for stable TDR strain sensing under complex environmental conditions, we contrast the distributed-parameter equivalent electromagnetic behaviors of the parallel two-conductor architecture with a single-conductor line (**Figure S2**). The parallel twin-line configuration forms a self-contained, balanced two-port transmission line network, where one LM channel conveys the forward pulse and the adjacent parallel line acts as a dedicated, low-impedance ground return path (**Figure S2A**). Because the electromagnetic field is tightly confined within the dielectric elastomer encapsulation, signal propagation is strictly governed by the intrinsic line parameters—loop inductance (*L*_0_) and line-to-line capacitance (*C*_0_)—yielding a phase velocity $v=1/\sqrt{\mu\varepsilon}=c/\sqrt{\varepsilon_{r}}$ that depends exclusively on the relative permittivity (*ε*_r_) of the silicone matrix. This structural encapsulation confers absolute immunity to external proximity interference, establishing a deterministic, linear mapping between time delay (Δ*t*) and physical length (*L*).

**Figure S2** Equivalent circuit model of the **A** Parallel two-line and **B** Single-line.

In stark contrast, an isolated single-conductor line lacks a physical return topology and is forced to couple with surrounding environmental objects (*e.g.*, ground planes, laboratory benches, metal frames, or human bodies) via distributed parasitic capacitances (*C*_para_) to complete its electrical loop (**Figure S2B**). During high-frequency pulse excitation, displacement currents (*i*_d_=*C*_para_⋅d*V*/d*t*) leak through these unpredictable external capacitive paths, forming an unbalanced, open propagation system with a dynamically fluctuating effective permittivity (*ε*_eff_). Consequently, minor ambient movements, relocations across heterogeneous surfaces, or mechanical deformations dynamically reconfigure the parasitic capacitive network, inducing severe signal distortion, velocity jitter, and erroneous displacement readouts.

Experimental validations confirm that while external conductor proximity (0–10 cm metal stick approach) and substrate repositioning induce pronounced waveform shifts and strain errors in the single-conductor line (**Figure S3A,B**). In stark contrast, the parallel two-conductor structure maintained exceptionally stable, deterministic responses (**Figure S3C,D**).

Furthermore, when the single-conductor sensor was stretched in 5 cm step increments, the measured deformation deviated noticeably from the applied values and exhibited substantial fluctuations (**Figure S3E,F**). This further demonstrates the severe limitations in accuracy and reliability of the single-line design for quantitative strain sensing.

**Figure S3.** **A** Reflection waveforms of a single LM conduit under external metal-conductor proximity at varying distances (0–10 cm). **B** Reflection waveforms of a single liquid-metal conduit at different positions on a laboratory workbench. **C** Reflection waveforms of dual liquid-metal conduits under external metal-conductor proximity at varying distances (0–20 cm). **D** Reflection waveforms of dual liquid-metal conduits at different positions on a laboratory workbench. Reflected waveforms of a single liquid-metal conduit under step-wise elongation. Corresponding reflection-delay changes and measured elongations under applied stretch.

**Supporting Information Note S2: Benchmarking against state-of-the-art soft TDR sensing platforms**

While conventional amplitude-based strain sensors (piezoresistive, capacitive, or inductive) suffer from severe cross-axis mechanical crosstalk and complex calibration requirements, TDR offers a paradigm shift by directly measuring the absolute, physical wave propagation delay (Δ*t* = 2*L*/*v*). Within the specialized domain of soft and stretchable TDR sensing, previous pioneering studies have primarily targeted multi-segment deformation identification or spatial touch localization by executing multi-peak tracking and complex signal reconstruction algorithms to solve inverse scattering problems (**Table S1**)[1-3].

In contrast, our parallel twin-conductor LM–TDR platform focuses on intrinsic, global axial strain decoupling. By establishing a uniform differential transmission line architecture, our approach bypasses heavy matrix-reconstruction algorithms entirely. Instead, an algorithmically lightweight differential peak-detection workflow directly extracts the terminal reflection's time-of-flight shift (Δ*t*), achieving linear, calibration-free strain tracking with complete immunity to non-axial mechanical crosstalk (**Table S1**).

**Table S1** Comparison of representative soft TDR sensing platforms

| **Feature** | **Soft LM TDR[1]** | **Multisegment TDR[2]** | **CMOS-integrated TDR[3]** | **This work** |
| --- | --- | --- | --- | --- |
| **Primary target** | Distributed Multimodal Localization | Multi-segment strain sensing | Integrated, low-power readout | Intrinsic axial strain decoupling |
| **Line architecture** | LM fibers | Segmented soft conduits | On-chip integrated line | Twin-conductor stretchable conduit |
| **Deformability** | Stretchable | Stretchable | Stretchable | Stretchable |
| **Signal processing algorithm** | Complex impedance discontinuity reconstruction | Multi-peak spectral analysis | Threshold-based peak detection | Direct terminal edge-fitting (Δ*t* extraction), algorithm-free |
| **Off-axis crosstalk immunity** | Not evaluated (focuses on local pressure) | Moderate (susceptible to inter-segment coupling) | Moderate | Evaluate immunity to compression, bending, biaxial stretching, and scratch |
| **Calibration requirement** | Complex multi-parameter mapping | Segment-by-segment calibration | Baseline calibration required | Self-calibrated |

**Supporting Information Note S3: Fundamental paradigm shift and architectural advancements over frequency-domain phase-delay sensing architecture**

To establish the novelty and performance advancements of the present TDR sensing platform, we contrast its governing physical principles with our previous frequency-domain phase-delay framework[4] The primary breakthrough lies in transitioning from a frequency-domain phase integration mechanism to a time-domain edge reflection paradigm (**Figure S4**).

**Figure S4 A** Previous single-conductor platform[4]: A continuous sinusoidal wave propagates along a two-port single conductor requiring physical connections at both ends and an auxiliary reference line. Physical length (Δ*L*) is mapped into an integrated scalar phase delay (Δ*ϕ*), making the overall readout vulnerable to multi-point local perturbations and external proximity interference. **B** Present single-ended parallel twin-line TDR platform: An ultra-fast square pulse is injected into a self-contained twin-line structure, directly resolving the discrete round-trip time-of-flight (Δ*t* = 2*L*/*v*).

In our previous work[4], a continuous sinusoidal wave traveled along a two-port single conductor, where physical length (*L*) was mapped into a continuous, accumulated phase delay (Δ*Φ* = 2π*fL*/*v*). Although functional, this approach compresses spatial variations into a globally integrated scalar value, making it vulnerable to multi-point local perturbation errors, while requiring physical connections at both sensor ends alongside an external reference line for differential phase detection (**Figure S4A**).

In stark contrast, the present work injects an ultra-fast square pulse into a parallel twin-line transmission line and directly measures the discrete round-trip time-of-flight (Δ*t* = 2*L/v*). This fundamental transition from frequency-domain phase tracking to time-domain edge reflection yields three crucial architectural advancements (**Figure S4B**): First, it enables a single-ended connection topology where signal injection and echo collection occur strictly at the proximal port, eliminating far-end electrodes or long return routings for seamless integration. Second, it naturally decouples multi-point local perturbations; any localized impedance mismatch at a distance *d* merely generates a minor, transient echo at its specific timestamp (*t* = 2*d/v*), leaving the terminal open-circuit reflection arrival time (*t* = 2*L*/*v*) completely uncompromised. Third, by replacing the open single-conductor configuration with a self-contained parallel twin-line structure, the electromagnetic field is tightly localized within the dielectric encapsulation. This confers intrinsic environmental immunity without relying on external hardware compensation or auxiliary reference lines, ultimately delivering superior stability, simplified system architecture, and uncompromised length-sensing fidelity under complex physical states. To explicitly delineate the fundamental novelty and structural superiority of this work, a comprehensive side-by-side comparison between the two sensing paradigms is summarized in **Table S2**.

**Table S2** Comparison of sensing mechanisms and key characteristics between Ref.[4] and this work

| **Feature** | **Ref. [41]** | **Present work** | **Key advancement** |
| --- | --- | --- | --- |
| **Sensing mechanism** | Frequency-domain phase accumulation | Time-domain reflection | Decouples local perturbations |
| **Excitation signal** | 15 MHz Sinusoidal wave | 50 kHz square wave | Simpler signal generation; immunity to carrier phase drift |
| **Port topology** | Two-port  (transmission mode) | Single-ended  (Reflection mode) | Eliminates far-end wiring and long return lines |
| **Reference channel** | Required | Not required | Lower hardware complexity |
| **Calibration requirement** | Initial phase baseline calibration | Velocity calibration via initial time delay | Simplified calibration |
| **Spatial resolution** | ~5 mm | 10 mm (standard) | Comparable resolution |

**Figure S5** **A** Fabrication procedure for the LM–filled latex elastomer conduit. The latex tube (cut to the desired length) was filled by syringe injection. Tensile stress–strain curve of the latex conduit, showing an elongation at break of 500% and an elastic modulus of 1 MPa. **B** Assembly and mechanical integrity of the parallel-wire configuration. Two LM-filled latex conduits were aligned side-by-side and bonded with a thin layer of latex solution serving as an elastomeric adhesive matrix, forming a mechanically integrated dual-conduit structure. No interfacial delamination was observed even when the structure was stretched to 300% tensile strain.

**Figure S6** **A** Incident voltage pulse waveform recorded by the oscilloscope at the output of the pulse generator without the LM transmission line connected. **B** Voltage pulse waveform recorded after connecting the parallel-wire LM transmission line, showing both the incident signal and the reflected signal from the open-circuit end of the LM conduit.

**Figure S7 Reflected waveforms at different pulse frequencies.** Regarding the internal frequency, our setup employs a 50 kHz square wave as the default repetition rate. It should be emphasized that the temporal resolution of TDR is determined by the high-frequency spectral components within the pulse rise time and bandwidth, whereas the pulse repetition frequency can be flexibly chosen over a broad operating range without compromising spatial precision.

At high frequencies, the upper operating limit is predominantly constrained by multi-reflection signal degradation. As the frequency increases (typically above 10 MHz for our transmission lines), residual high-order reflections from previous cycles fail to attenuate completely and superimpose onto the incoming primary pulse of the next cycle, causing waveform distortion and measurement error.

At low frequencies, there is no fundamental physical lower limit. Lowering the repetition rate simply reduces the temporal data update density per unit time without affecting the physical accuracy of the time-of-flight measurement. When applying 16-frame waveform averaging, even a low repetition rate of 800 Hz achieves a rapid refresh interval of 20 ms (16/800 Hz=0.02 s), which is fully sufficient for real-time motion tracking. Our selected default frequency of 50 kHz delivers an even faster effective refresh time of 0.32 ms (16/50 kHz=0.00032 s), effortlessly eliminating human-perceptible latency while remaining well below the high-frequency multi-reflection interference threshold.

Consequently, our system operates stably and reliably within an optimal TDR interrogation frequency window below 10 MHz (defaulted at 50 kHz for standard laboratory characterization).

**Figure S8** Objective and automated extraction workflow for the round-trip propagation time delay.

To eliminate subjective manual selection bias, the time delay is extracted via an automated differential peak-detection algorithm. For each experimental state, 16 consecutive waveforms are acquired and point-by-point averaged directly by the oscilloscope to suppress random noise. A two-point interval difference

$$\Delta V\left[ i \right]=V\left[ i+1 \right]-V\left[ i-1 \right]$$

where V[i] represents the averaged voltage at the $i$-th sampling point is then calculated, transforming the sharp rising edges of the incident pulse and the open-circuit terminal reflection into two distinct positive local peaks. By executing a peak-search algorithm within preset time windows, the arrival timestamps corresponding to the incident edge (*t*_1_) and the terminal reflection edge (*t*_2_) are automatically identified, and the round-trip propagation time delay is precisely calculated as Δ*t* = *t*_2_-*t*_1_.

**Figure S9** Reflection signals measured for LM transmission lines with different conduit diameters but identical lengths, showing negligible variation in time-of-flight. The inset schematically illustrates the geometric parameters used to define the conduit for comparison.

**Figure S10 Propagation velocity extracted from LM conduits with different dimensions under 0% strain.** **A** Representative reflected waveforms measured for conduits of different lengths. **B** Linear fitting of reflection delay as a function of line length, yielding a consistent propagation velocity of approximately 1.96 × 10^8^ m/s.

**Figure S11** **Reflected signals of LM transmission lines fabricated with different elastomeric materials.** **A** Silicone tubing. **B** Ecoflex 00-30. These demonstrate consistent strain-sensing behavior across different materials.

**Figure S12** **Schematic illustration of bending effects on time-delay sensing. A** Bending-induced axial elongation when mounted away from the neutral axis. **B** Natural bending and localized folding without axial elongation.

When the sensor is conformally mounted onto the outer surface of a bending substrate, its positioning away from the structural neutral axis forces the LM line to stretch along the outer perimeter, as illustrated in **Figure S12A**. In this configuration, the detected Δ*t* shift is generated directly by the bending-induced physical axial elongation rather than the localized bending curvature itself. The arc length of the outer surface is given by

$$L_{\mathrm{outer}}=(R+d/2)\text{ }\theta$$

where *d* is the thickness of the strip. The resulting elongation of the outer surface relative to the neutral axis is therefore

$$\Delta L=L_{\mathrm{outer}}-L_{0}=\theta\text{ }(d/2)$$

In other case, when the sensor undergoes arbitrary spatial routing, local bending, or folding in free space—where the conductor remains along its structural neutral axis—the net change in total electrical path length is negligible, as illustrated in **Figure S12B**. In this scenario, local impedance variations may induce slight localized waveform ripples, but the overall round-trip propagation delay remains completely unchanged. This unique property proves that the sensor can be conformally deployed across sharp corners, intricate surface geometries, or mobile structural joints without inducing false strain readings.

**Figure S13** **Insensitivity of the time-of-flight delay to off-axis perturbations and temperature under axial stretching.** **A** Under stepwise axial tensile stretch with superimposed localized transverse compression (0–80%). **B** Under stepwise axial tensile stretch with superimposed localized folding at different angles (0–150°). **C** Under stepwise axial tensile stretch at different temperatures (20–80 °C). In all cases, the delay is governed primarily by axial elongation, confirming minimal sensitivity to compression, folding, transverse strain, and temperature.

**Figure S14** **Response of the LM-TDR sensor to uniaxial elongation under multiple localized perturbations.** **A** No off-axial deformation. **B** Two localized compressive points applied along the conduit. **C** Three localized compression points; **D** One bending point combined with two compression points. In all cases, the reflection delay varies linearly with the applied axial elongation, demonstrating that the length measurement is unaffected by the number or type of localized deformations.

**Figure S15** **Reflection responses of an LM conduit subjected to localized perturbations. A** For applied local compressive strains ranging from 0% to 80%, the reflection signal originating from the open-circuit end remains essentially unchanged, indicating that localized deformation does not influence the effective electrical propagation length of the conduit. However, when the compression becomes sufficiently large to completely pinch off the LM channel, the reflection peak shifts abruptly, corresponding to the formation of an open-circuit condition at the compressed site. **B** Reflection responses under localized folding-induced deformation, with the signal exhibiting similar behavior to the compression case, shifting only when the folding introduces an open-circuit condition.

**Supporting Information Note S4. Influence of transverse strain on characteristic impedance and time-delay fidelity**

Transverse stretching alters cross-sectional dimensions—such as the LM channel radius (*a*) and inter-conductor spacing (*D*)—thereby modulating the local characteristic impedance (*Z*_0_), reflection coefficients (*Γ*), and local waveform amplitudes during multiaxial deformation. To evaluate whether these amplitude variations affect time-delay extraction accuracy, we provide both a transmission line theoretical analysis and experimental validation under localized non-uniform transverse deformations.

For the parallel twin-line transmission line embedded in a dielectric elastomer, the characteristic impedance (*Z*_0_) is given by:

$Z_{0}\approx\frac{1}{\pi}\sqrt{\frac{\mu}{\varepsilon}}\cosh^{-1} \left( \frac{D}{2a} \right)$

where *a* is the LM channel radius, *D* is the center-to-center conductor distance, *μ* is the magnetic permeability, and *ε* is the dielectric permittivity of the encapsulation layer.

Transverse stretching varies *a* and *D*, thereby changing *Z*_0_ and the local reflection coefficient *Γ*. However, for a low-loss transmission line operating under the dominant TEM mode, the phase velocity *v* is strictly defined as:

$v=\frac{1}{\sqrt{\mu\varepsilon}}=\frac{c}{\sqrt{\varepsilon_{r}}}$

Crucially, the propagation velocity *v* depends exclusively on the intrinsic electromagnetic properties of the dielectric substrate (*μ*, *ε*) and is entirely independent of the cross-sectional geometric parameters (*a* and *D*).

When the entire transmission line experiences **uniform transverse strain**, *Z*_0_ shifts uniformly along the line. Because *v* remains unchanged and no internal impedance discontinuities are created, the round-trip propagation time delay (Δ*t*) remains unaffected (see **Figure 3C** in the manuscript). When transverse deformation is **non-uniform strain**, the transmission line can be discretized into multiple segments with different local impedances (*Z*_0, i_). At the interface between two adjacent segments, a local reflection coefficient *Γ*_i_ is generated:

$\Gamma_{i}=\frac{Z_{0,i+1}-Z_{0,i}}{Z_{0,i+1}+Z_{0,i}}$

These local impedance mismatches produce small intermediate reflections. However, because *v*_i_=*v* remains constant across all segments, the total round-trip time delay for the primary terminal open-circuit reflection is strictly cumulative:

$\Delta t_{\text{terminal}}=\sum_{i} \frac{2L_{i}}{v_{i}}=\frac{2\sum L_{i}}{v}=\frac{2L_{\text{total}}}{v}$

As long as localized impedance mismatches do not cause complete signal extinction (*e.g.*, an open or short circuit), a sufficient portion of the electromagnetic pulse transmits through to the open-circuit terminal. Because intermediate local reflections arrive earlier in time, our windowed differential edge-detection algorithm easily isolates the primary terminal reflection peak.

Consequently, transverse-strain-induced amplitude variations introduce zero time-delay error, ensuring absolute measurement fidelity of physical length.

**Figure S16** **Waveforms and time-delay responses under biaxial loading of axial stretch (x-direction) combined with transverse stretch (y-direction).** **A** Reflected waveforms under non-uniform transverse stretching. **B** Measured time delays under axial stretch combined with non-uniform transverse stretch, showing that transverse deformation produces negligible influence on the delay. **C** Measured time delays under axial stretch combined with uniform transverse stretch, showing similarly negligible influence on the delay. As shown in **Figure S16**, despite the localized cross-sectional distortion and non-uniform impedance variations, the TDR reflection waveforms and extracted terminal time delays (Δ*t*) remain virtually overlapping (terminal delay deviation < 2.1%). This confirms that non-uniform transverse stretching neither alters the signal propagation speed nor degrades terminal open-circuit detection, validating the high stability and robustness of our TDR sensing platform under complex multiaxial deformation.

**Figure S17** Infrared thermal images of the sensor acquired at different combinations of tensile strain and temperature.

**Supporting Information Note S5. Performance of LM conduits under localized physical scratches and leakage**

While physical scratches and LM leakage represent a common, intrinsic challenge for stretchable electronics, our TDR-based sensing platform exhibits a unique electromagnetic response mechanism that distinctly differentiates damage states from normal strain signatures. To establish its operational boundaries, we categorize localized scratches into three physical scenarios based on channel continuity, leakage severity, and inter-conductor bridging, and systematically analyze their governing transmission-line equivalent models alongside experimental validations (**Figure S18**).

**Figure** **S18** **Reflected waveforms under local damage.** **A** Minor leakage with preserved electrical continuity. **B** Progressive loss of electrical continuity. **C** Leaked LM bridging short circuit.

**(1) Minor scratch with slight LM leakage, where the conductive path remains continuous**

When the elastomeric encapsulation is locally ruptured but the internal LM remains continuous without severe cross-sectional necking (**Figure S18A, inset**), the damage site is modeled as a localized non-uniform transmission line segment characterized by an altered characteristic impedance *Z*_d_

$Z_{d}=\sqrt{\frac{L_{d}}{C_{d}}}$

Where *L*_d_ and *C*_d_ represent the localized parasitic inductance and capacitance changes caused by geometric deformation at the damage site.

When the signal enters and exits the damaged region, local reflections are generated. The interface reflection coefficient can be written as:

$\Gamma=\frac{Z_{d}-Z_{0}}{Z_{d}+Z_{0}}$

Because the damaged region is typically short, the two reflections generated at its entry and exit points merge in the time domain into a small, localized waveform perturbation. Although this reflection arrives earlier than the open-circuit terminal echo, as long as the damage remains small and conductive continuity is maintained, the local reflection is weak, and sufficient transmitted signal passes through, allowing the electromagnetic wave to continue propagating to the true open-circuited far end. Consequently, the primary open-circuit reflection from the far end is still captured, and its arrival time remains determined by the total electrical length of the line.

As shown in **Figure S18A**, when a minor scratch occurs without connecting the two conductors together, the reflection curve is virtually identical to that of the intact state, perfectly matching our theoretical analysis.

**(2) Severe leakage leading to conductor necking or open circuit**

As LM leaks extensively under severe mechanical damage, the local cross-sectional area decreases markedly, inducing necking or complete path interruption. Modeling this damage as a localized series impedance *Z*_s_, the reflection and transmission coefficients become:

$\Gamma=\frac{Z_{s}-Z_{0}}{Z_{s}+Z_{0}}$

$T=\frac{2Z_{0}}{2Z_{0}+Z_{s}}$

As *Z*_s_ increases, the reflection generated at the damage location intensifies, while the signal continuing toward the true far end diminishes. Since the terminal reflection must pass through the damaged site twice (on both the forward and return paths), its amplitude undergoes an attenuation factor proportional to *T*^2^. When the damage approaches a complete open circuit (*Z*_s_→∞), we have *Γ* → 1 and *T* → 0. Under this condition, virtually the entire signal is reflected back at the damage location. The transmission line beyond the damaged site is no longer observable by TDR, and the instrument measures the location of the damage rather than the full physical length of the sensor.

As shown in **Figure S18B**, as leakage increases, the reflection curves diverge progressively from the intact state, with the amplitude of the terminal reflection edge gradually diminishing. It should be noted that, once the electrical connection at the damaged location is fully broken, the primary reflection peak shifts forward to the damage timestamp.

**(3) LM bridging the twin conductors, giving rise to a short circuit**

Another severe damage case occurs when both LM channels are ruptured simultaneously, allowing extruded LM to form a conductive bridge between the two conductors. In this scenario, the damage location should be modeled as a local shunt/bridging resistance (*R*_b_) connected across the parallel lines, rather than a series impedance. The corresponding voltage reflection coefficient (*Γ*) and transmission coefficient (*T*) are expressed as:

$\Gamma=\frac{-Z_{0}}{2R_{\text{b}}+Z_{0}}$

$T=\frac{2R_{\text{b}}}{2R_{\text{b}}+Z_{0}}$

When *R*_b_ is relatively large, a portion of the signal still passes through the damaged site to reach the open end, though the terminal echo amplitude decreases. However, when the two conductors are almost completely short-circuited (*R*_b_ → 0), we have *Γ*→ -1 and *T*→ 0. Here, the differential-mode signal is completely reflected back at the short-circuit point with an inverted polarity, hiding the true open terminal. Consequently, the TDR system localizes the short-circuit point rather than measuring the overall sensor length.

As shown in **Figure S18C**, when both conductors are damaged simultaneously and fully connected together, the reflection curve changes dramatically from the intact baseline. A distinct negative reflection peak appears at the scratch location, and the original terminal reflection peak completely disappears.

In summary, minor scratches that maintain channel continuity have negligible impact on Δ*t* extraction and total strain measurement. Severe scratches causing open or short circuits will dominate the reflection waveform, shifting the echo timestamp to the damage location. Crucially, because these local damage echoes arrive earlier than the normal terminal echo and exhibit distinct polarities/shapes, they are easily distinguished from the true terminal reflection, preventing false strain readings.

**Figure S19** **Self-calibrated sensor length based on reflection waveforms.** **A** Sensor 1, with a self-calibrated initial length of 59 cm, according to the reflection time (Δ*t* = 6.031 ns) corresponding to the effective propagation length. **B** Sensor 2, with an initial length of 108 cm, according to the reflection time (Δ*t* = 11.062 ns). In each case, the reflection time directly corresponds to the initial conduit length, confirming the self-calibration process.

**Figure S20** **Self-calibrated sensor initial length and pre-strain determination from reflection waveforms.** **A** Determination of the initial conduit length from the round-trip delay (Δ*t* = 6.053 ns), corresponding to an absolute length of 59 cm. **B** Determination of an applied pre-stretch from the shifted reflection waveform. The additional delay (Δ*t* = 2.805 ns) corresponds to a pre-elongation of 27.5 cm, demonstrating that both absolute length and installed pre-strain can be directly quantified from the time-of-flight without external calibration.

**Figure S21 Cyclic loading–unloading performance at different strain levels.** **A–C** Reflected waveforms and corresponding cyclic time-delay/length responses over 10 loading–unloading cycles at 50%, 100%, and 200% strain, respectively. **D** Relative measurement errors at 50%, 100%, and 200% strain. **E** Residual length increments after unloading at different strains real-time tracked by our LM-conduit sensor.

**Figure S22 Flexible baseline definition enabled by self-calibration. A** Sensor response after prolonged holding at 100% strain for 24 h. A permanent elongation of 5.4 cm is observed as a residual set, manifested as a shift in the time-of-flight. Despite this irreversible deformation, the sensor accurately establishes a new baseline for subsequent measurements. **B** Sensor response after 200 cycles at 100% strain. A permanent elongation of 3.4 cm is observed as a residual set, manifested as a shift in the time-of-flight. Despite this irreversible deformation, the sensor accurately establishes a new baseline for subsequent measurements. **C** Baseline redefinition after cycling. By redefining the reference state following such conditioning histories, accurate relative elongation measurement is preserved under stepwise stretching, demonstrating stable performance independent of mechanical history.

**Supporting Information Note S6: Practical deployment flexibility, waveform overlap mechanism, and engineering strategies for short-segment strain sensing**

The proposed single-ended LM TDR sensor offers exceptional deployment flexibility, enabling customizable installation architectures tailored for complex wearable or robotic integration scenarios. For targeted, localized strain monitoring in spatially constrained areas (such as wearable finger joint tracking or micro-actuator sensing), the conduit can be conveniently routed such that only a compact 1-5 cm functional segment is attached to the high-strain region, while the upstream portion functions as an integrated interconnect line.

To systematically map the operational boundaries and deployment guidelines of this configuration, we investigate the fundamental pulse propagation behavior, the physics of waveform overlap in short lines, and the experimental lower length limit of the sensor platform.

**Figure S23 Schematic showing the waveform overlap in ultra-short conduits.** **A** Ideal TDR waveform with infinitely steep step edges, allowing the incident and terminal reflection edges to be clearly distinguished. **B** Practical TDR waveform with broadened edges caused by transmission loss and dispersion, leading to waveform overlap in short transmission lines.

For an ideal step signal with an infinitely steep vertical rising edge, the incident edge and the open-circuit terminal reflection can theoretically be distinguished on the time axis as long as the transmission line length is strictly non-zero (**Figure S23A**). However, in practical physical systems, the square wave’s rising edge is composed of a finite band of high-frequency harmonics. As the signal propagates along the LM transmission line, conductor attenuation, dielectric loss, and high-frequency dispersion progressively dampen these upper harmonics, smoothing the steep edge into a transition zone with a finite rise time (**Figure S23B**).

Because the open-circuit terminal reflection undergoes both forward and backward propagation, it suffers twice the channel attenuation. Consequently, the reflected edge is broader and more dispersed than the incident edge. For the long transmission lines, the round-trip time delay (Δ*t* = 2*L*/*v*) is significantly larger than the edge transition duration. The incident edge completes its transition before the terminal reflection arrives, leaving a clear intermediate voltage plateau between the two transitions. This allows *t*_1_ and *t*_2_ to be extracted independently.

As *L* decreases, Δ*t* shrinks proportionally. When Δ*t* becomes comparable to or smaller than the edge transition width, the terminal reflection arrives before the incident edge has finished rising. The two smooth rising transitions merge and superimpose, causing the intermediate plateau to shrink and eventually disappear. Under this overlap condition, the oscilloscope displays a single, continuously broadened rising edge rather than two distinct transitions. Consequently, the minimum usable length limit is governed by the temporal overlap of the incident and reflected edges, which masks the individual waveform features required for delay extraction.

To determine the lower length limit under our current hardware configuration (50 kHz square wave, 5 GSa/s sampling rate), we fabricated parallel twin-line sensors with lengths decreasing from 20 cm down to 8 cm and evaluated their TDR waveforms under identical excitation and acquisition settings (**Figure S24**).

**Figure S24** Reflected and differential waveforms for transmission lines with different lengths.

For sensor lengths ≥10 cm, a clear, repeatable intermediate plateau remains visible in the raw TDR waveforms. The differential curve yields two distinct, well-separated slope peaks corresponding to *t*_1_ and *t*_2_, allowing stable time-delay extraction. When the length drops to 8 cm, the two slope peaks in the differential domain merge into a single peak, and the raw waveform exhibits a single broadened rising edge. Thus, 10 cm represents the experimental lower length limit for standalone straight-line sensors under our current signal processing and hardware setup.As LM leaks extensively under severe mechanical damage, the local cross-sectional area decreases markedly, inducing necking or complete path interruption. Modeling this damage as a localized series impedance *Z*_s_, the reflection and transmission coefficients become.

Although a standalone 1–5 cm line suffers from edge overlap under our current pulser/oscilloscope setup, deformation sensing in compact spatial areas is entirely feasible. In practical deployments, the transmission line can be broadly applied, with only a 1–5 cm localized segment attached to the target region to monitor deformation (**Figure S25, inset**), while the remaining portion functions purely as a lead-in interconnect line. This approach effectively resolves space-constraint challenges during sensor installation.

**Figure S25** Reflected waveforms for 1 cm stepwise stretching of the terminal 5 cm segment

To validate this approach, we tested a 120 cm transmission line where only a 5 cm terminal segment was subjected to deformation, while the remaining 115 cm baseline line served as an interconnect line (as schematically illustrated in **Figure S25**). When stretching this 5 cm local segment in 1 cm steps, the terminal reflection peak shifts smoothly and reproducibly along the time axis. This proves that our TDR sensing approach can be readily installed and used to accurately measure deformations even within a 1–5 cm tight spatial constraint.

**Figure S26 Relative errors between sensor-derived elongation and tape-measure reference measurements in practical demonstrations.** **A** Lantern. **B** Balloon. **C** Artificial muscle. **D** Arm. **E** Leg. **F** Tourniquet.

**Figure S27** **Circumferential expansion of a collapsible lantern with localized rib-induced compression.** During unfolding (left to right), the lantern expands radially, generating coupled curvature and circumference changes. The supporting ribs create localized contact points that apply compressive forces to the LM conduit along the surface, illustrating nonuniform mechanical perturbations during curved-surface deployment.

**Figure S28** **Biaxial deformation of an inflatable balloon.** Two orthogonally arranged LM–TDR conduits are attached to the balloon surface. Upon inflation, the membrane undergoes approximately equiaxial expansion, inducing tensile strain along two orthogonal directions simultaneously. Each sensor experiences strain along its respective axis, enabling independent measurement of directional elongation.

**Supporting Information Note S7: Electromagnetic crosstalk analysis and simultaneous multi-channel acquisition protocol**

To evaluate potential electromagnetic interference between the orthogonally arranged LM–TDR channels used for biaxial strain sensing (**Figure 5C**), we systematically analyzed the coupling physics and quantified inter-channel crosstalk at the crossover intersection.

When high-frequency excitation pulses propagate along an active channel (aggressor), high-frequency edge transitions (d*V*/d*t* and d*I*/d*t*) can theoretically induce transient coupling into an adjacent channel (victim) via mutual capacitance (*C*_m_) and inductance (*L*_m_). However, for orthogonally intersecting lines, coupling is strictly localized to the physical crossover point as a discrete lumped element, rather than accumulating along an extended length. Crucially, because the signal propagation vectors and high-frequency magnetic field lines of the two channels are mutually perpendicular (*E*_A_⊥*E*_B_), mutual inductive coupling is physically minimized, preventing continuous interference.

**Figure S29** Reflected waveforms under inter-channel crosstalk.

As shown in **Figure S29**, localized coupling, two identical LM–TDR channels were arranged orthogonally and operated under simultaneous 50 kHz excitation while varying the vertical separation distance at the intersection (d = 0 cm to 10 cm). Across all tested spacings—even at direct physical contact (d = 0 cm)—inter-channel crosstalk remained virtually undetectable. The primary reflection timestamps and time-of-flight (Δ*t*) extractions of Channel B were completely unaffected by signal propagation in Channel A.

Consequently, both orthogonal channels in the biaxial sensing platform (**Figure 5C**) are operated and acquired **simultaneously**. The intrinsic immunity of orthogonal geometry eliminates the need for complex, time-sequential multiplexing hardware, enabling rapid, multi-axis strain tracking without crosstalk degradation.

**Figure S30** **Pneumatic artificial muscle under pre-strain and actuation.** The LM–TDR sensor is mounted on the artificial muscle in the relaxed (unpressurized) state, where it is already under substantial pre-strain due to installation. Upon actuation (pressurization), the muscle contracts axially, shortening the overall length. This configuration illustrates sensor operation under sustained pre-strain and dynamic contraction.

**Figure S31** **Dynamic performance of the sensor under elastic band loading. A** Schematic and experimental setup of the dynamic test using elastic resistance bands during repetitive exercise. **B** Comparison of peak elongation measured by the sensor and a commercial force gauge over multiple loading cycles, showing strong quantitative agreement and stable signal capture under sustained pre-strain and dynamic loading conditions.

**Figure S32** Tourniquet tightening process.

**Figure S33** Force–strain curve of the elastic tourniquet**.**

Our proposed sensor operates on the well-established principles and methodology of TDR. Consequently, its excitation, measurement, and future deployment can readily draw upon the rich heritage of conventional rigid-line TDR systems, which are already highly mature and widely used.

Currently, TDR signal generation, high-speed reflection acquisition, and propagation delay calculations do not inherently depend on bulky benchtop instruments. Instead, they are routinely integrated into compact, handheld, or modular hardware architectures. For instance, representative commercial TDR systems—spanning handheld cable fault locators, compact modular units, and high-precision field instruments—are summarized in **Table S3**. These commercial implementations convincingly demonstrate that sub-nanosecond pulse generation, reflection signal acquisition, and automated delay extraction possess a solid engineering foundation for miniaturization.

**Table S3:** Commercial TDR systems demonstrating hardware miniaturization and integration capability

| **Category** | **Hardware specifications** | **Spatial resolution** | **Practical portability** |
| --- | --- | --- | --- |
| **Radio-detection**  **1205CXB** | 1 GSa/s sampling rate  3 ns pulse width  1–16 waveform averaging | 2.5 cm | Handheld, battery-powered cable tester |
| **Megger**  **TDR2050** | 2 ns pulse width  dual-channel trace | 10 cm | Compact handheld unit |
| **PicoScope**  **9311-20** | 60 ps transition time  integrated pulse generator | 4 mm | Ultra-compact USB/PC-based module |
| **Teledyne LeCroy**  **WavePulser 40iX** | High-bandwidth pulser with TDR analysis | <1 mm | Integrated modular instrument |

**References**

[1]	A. Leber, C. Dong, R. Chandran, et al., “Soft and Stretchable Liquid Metal Transmission Lines as Distributed Probes of Multimodal Deformations,” *Nature Electronics* 3, no. 6 (2020): 316-326. https://doi.org/10.1038/s41928-020-0415-y.

[2]	C. Wilson, F.-C. Moldovan, and M.L. Johnston, “Real-Time Stretchable Strain Sensor using CMOS-based Time-Domain Reflectometry,” in *2025 IEEE Sensors* (2025): 1-4. https://doi.org/10.1109/SENSORS59705.2025.11330537.

[3]	C. Wilson and M.L. Johnston, “Multi-segment Stretchable Strain Sensor using Time Domain Reflectometry,” in *2024 IEEE International Symposium on Circuits and Systems* (2024): 1-5. https://doi.org/10.1109/ISCAS58744.2024.10558477

[4]	Y. Zeng, Y. Gao, B. Yao, et al., “Geometry-Defined Tensile Deformation Sensing via Electromagnetic Phase Delay in a Liquid-Metal Conduit,” *Applied Physics Letters* 128 (2026): 082701. https://doi.org/10.1063/5.0319988
